# Identification and mapping of expressed genes associated with the 2DL QTL for fusarium head blight resistance in the wheat line Wuhan 1

**DOI:** 10.1186/s12863-019-0748-6

**Published:** 2019-05-21

**Authors:** Xinkun Hu, Hélène Rocheleau, Curt McCartney, Chiara Biselli, Paolo Bagnaresi, Margaret Balcerzak, George Fedak, Zehong Yan, Giampiero Valè, Shahrokh Khanizadeh, Thérèse Ouellet

**Affiliations:** 1 Ottawa Research and Development Centre, Agriculture and Agri-Food Canada, 960 Carling Ave, Ottawa, ON K1A 0C6 Canada; 2 Triticeae Research Institute, Sichuan Agricultural University, 211 Huimin Road, Wenjiang, Chengdu, Sichuan 611130 People’s Republic of China; 3 Morden Research and Development Centre, Agriculture and Agri-Food Canada, 101 Route 100, Unit 100, Morden, Manitoba R6M 1Y5 Canada; 4 CREA, Council for Agricultural Research and Economics - Research Centre for Genomics and Bioinformatics, Via S. Protaso 302, I-29017 Fiorenzuola d’Arda, PC Italy; 5 Dipartimento di Scienze e Innovazione Tecnologica, Università del Piemonte Orientale, Vercelli, Italy

**Keywords:** Wheat, Wuhan 1, Fusarium head blight, RNA-Seq, Expression QTL, 2DL FHB resistance QTL

## Abstract

**Background:**

*Fusarium* head blight (FHB) is a problem of great concern in small grain cereals, especially wheat. A quantitative trait locus (QTL) for FHB resistance (FHB_SFI) located on the long arm of chromosome 2D in the spring wheat genotype Wuhan 1 is a resistance locus which has potential to improve the FHB resistance of bread wheat since it confers effective resistance to wheat breeding lines. Recently, differentially expressed genes (DEG) have been identified by comparing near isogenic lines (NIL) carrying the susceptible and resistant alleles for the 2DL QTL, using RNA-Seq. In the present study, we aimed to identify candidate genes located within the genetic interval for the 2DL QTL for FHB resistance, as assessed by single floret inoculation (FHB_SFI), and possibly contributing to it.

**Results:**

Combining previous and additional bioinformatics analyses, 26 DEG that were located on chromosome arm 2DL were selected for further characterization of their expression profile by RT-qPCR. Seven of those DEG showed a consistent differential expression profile between either three pairs of near isogenic lines or other genotypes carrying the R and S alleles for the 2DL QTL for FHB resistance. *UN25696*, which was identified in previous expression work using microarray was also confirmed to have a differential expression pattern. Those eight candidate genes were further characterized in 85 lines of a double haploid mapping population derived from the cross Wuhan 1/Nyubai, the population where the 2DL QTL was originally identified. The expression QTL for gene *Traes_2DL_179570792* overlapped completely with the mapping interval for the 2DL QTL for FHB_SFI while the expression QTL for *UN25696* mapped near the QTL, but did not overlap with it. None of the other genes had a significant eQTL on chromosome 2DL. Higher expression of *Traes_2DL_179570792* and *UN25696* was associated with the resistant allele at that locus.

**Conclusions:**

Of the 26 DEG from the 2DL chromosome further characterized in this study, only two had an expression QTL located in or near the interval for the 2DL QTL. *Traes_2DL_179570792* is the first expression marker identified as associated with the 2DL QTL.

**Electronic supplementary material:**

The online version of this article (10.1186/s12863-019-0748-6) contains supplementary material, which is available to authorized users.

## Background

Bread wheat (*Triticum aestivum* L.) is one of the three most widely grown cereals worldwide, contributing to about 20% of the food calories eaten by humans. Fusarium head blight (FHB), also called scab or head scab, is a devastating fungal disease of wheat, with frequent outbreaks in warm and humid or subhumid regions worldwide. More than 17 *Fusarium* species can cause FHB on wheat, while *Fusarium graminearum* Schwabe (*Hypocreales*: *Nectriaceae*) is the most virulent *Fusarium* species [1]. FHB can cause serious yield lost through shriveled kernels, and reduce the milling, baking and pasta-making quality of the grain [2–4]. However, the most serious hazard caused by FHB is the contamination of seeds with toxic fungal secondary metabolites called mycotoxins, including deoxynivalenol (DON) and its derivatives; these render the seeds unsuitable for human or animal consumption [1, 5–7]. Although fungicide application can partly control the disease, their use increases the cost of wheat production and contaminates the environment. Genetic improvement of wheat for increased resistance to FHB is an economical and environment-friendly strategy to control FHB.

Many sources of genetic resistance to FHB have been documented from wheat and its relatives [8–14], and mapping studies have shown that QTL for FHB resistance were distributed on all wheat chromosomes [1]. The most widely used source of genetic resistance in wheat breeding programs is Sumai 3 and its derivatives; two FHB resistance genes, *Fhb1* and *Qfhs.ifa-5A*, have been repeatedly mapped, on chromosomes 3BS and 5A respectively, in material derived from that source [1, 15]. *Fhb1* is associated with type II resistance, a reduction in spreading within the spike, while *Qfhs.ifa-5A* is associated with type I resistance, a reduced initial infection. Very recently, the gene encoded by *Fhb1* has been identified as a chimeric lectin [16]; this is the very first gene corresponding to a FHB resistance QTL that has been identified.

It is believed that type II resistance is less affected by environmental variability and can confer a more stable resistance phenotype. Numerous type II resistance QTL have been identified from chromosomes 1BL [17], 2A [18], 2B [19], 2DL [20, 21], 3BS and 6BS [22–24].

In recent years, transcriptomic studies using microarray [25–27] and next generation RNA sequencing (RNA-Seq) [28–30] have become effective genomics strategies to identify differentially expressed genes (DEG) between FHB-resistant (R) and -susceptible (S) wheat genotypes, and suggest molecular mechanisms to explain the resistance.

Somers et al. [20] crossed Wuhan 1 with Nyubai, both moderately resistant to FHB, to develop a double haploid (DH) population, and identified a type II resistance QTL for FHB on chromosome arm 2DL of Wuhan 1, in addition to QTL on 3BS and 5AS from Nyubai. Long et al. [31] carried out a gene expression comparison using microarray on a pair of near isogenic lines (NIL) derived from that original DH population and contrasting for the presence of the 2DL QTL; both NIL carried the S allele for the 3BS and 5AS QTL. The study identified eight genes differentially expressed in spikelets whose expression profile correlated either with the presence or absence of the 2DL QTL. Recently, an RNA-Seq experiment was conducted on the same pair of NIL, comparing both rachis and spikelet tissues among the two genotypes; a comprehensive transcriptomic analysis uncovered deployment of different defense strategies between the two NIL including those associated with sugar signaling [32]. A list of DEG was developed from that study, including genes with unique expression profiles associated with either the presence or the absence of the 2DL QTL. Still using the same pair of NIL contrasting for the 2DL QTL, a metabolomic study has led to the identification of a WRKY70 transcription factor and three biosynthetic enzymes as possible candidate genes contributing to the FHB resistance provided by the 2DL QTL [33]. The findings in Biselli et al. [32] and additional analyses in this study have led to the development of a list of DEG that are predicted to be physically located on the 2DL chromosome. In addition to the validation of a correlative relationship with the 2DL QTL for one of the candidate genes identified by Long et al. [31], eight additional candidate genes have been identified here. Using expression profiling in the original Wuhan 1/Nyubai DH mapping population, the expression QTL (eQTL) for one of those candidate genes was shown to overlap with the 2DL QTL for FHB resistance.

## Methods

### Plant materials

Seeds from three pairs of NIL, 2–2890 (2DL-) and 2–2618 (2DL+), 2–3251 (2DL-) and 2–3213 (2DL+), and 2–2674 (2DL-) and 2–2712 (2DL+), were kindly provided by Dr. Daryl Somers (Cereal Research Centre, Agriculture and Agri-Food Canada). The NIL were developed by crossing the FHB resistant genotype HC374 (male parent; pedigree = Wuhan 1/Nyubai; HC374 carried 5 FHB resistance QTL [20]) with the moderately susceptible variety CDC Alsask (female parent; formerly called BW301), and backcrossing twice with CDC Alsask as recurrent parent (Additional File 1). Foreground selection was used to retain the 2DL QTL and eliminate the four other FHB QTL (3BS, 3BSc, 4B, and 5AS). Background selection was also used to increase recovery of the ‘CDC Alsask’ background by using markers across the genome that are unlinked to the FHB QTL [34]. As each NIL pair traces back to a single BC2F1 plant, there is minimal residual variation within a pair. After BC_2_F_1_, the material was self-pollinated for four generations to generate the BC_2_F_5_ progeny used in this study. In each generation, the QTL identity of the NIL was confirmed by marker-assisted selection [35]; the 2DL QTL interval was defined by the presence of Wuhan 1 alleles for the SSR markers gpw5141, gpw8003, gwm539, gwm608, cfd73 and cfd233. In each pair, one NIL carried the R allele for the 2DL QTL while the other NIL carried the S allele at that locus; all 6 NIL carried the S allele for the QTL 3BS, 3BSc, 4B and 5AS described in [20]. One pair of NIL, 2–2890 and 2–2618, has previously been used to generate an RNA-Seq dataset [32]. Wuhan 1, Nyubai, HC374 and the FHB-susceptible cultivar Shaw were also used to characterize gene expression profiles. Eighty-five DH lines from the cross Wuhan 1/Nyubai [20] were used for eQTL analysis. Wheat plants were grown in controlled-environment cabinets with 16 h light at 20 °C and 8 h dark at 16 °C until mid-anthesis then transferred to growth chambers at anthesis. Plant growth conditions were described previously [36].

### *F. graminearum* inoculation and tissues sampling

A highly virulent and 15ADON producing isolate of *F. graminearum*, DAOM 233423 (Canadian Collection of Fungal Cultures, Agriculture and Agri-Food Canada, Ottawa, Canada), was used for infecting wheat. *F. graminearum* inoculum preparation and plant grow conditions were as described previously [36]. Twelve (for NIL, Wuhan 1 and Nyubai) and two (for DH lines) pots with three seedlings each were grown for each line. At mid-anthesis, ten μl of either a *F. graminearum* macroconidial spore suspension at 1 × 10^5^ spores/mL, or water (mock control), was point-inoculated with a micropipette between the lemma and palea of two basal florets of each fully developed spikelet on each treated head. Following inoculation, plants were transferred into a growth room where they were misted overhead; within each treatment, pots were disposed in a random order. Misting was for two days, 30 s every 1 h, during the light period. For the 3 pairs of NIL contrasting for the presence of the 2DL QTL, 6 to 7 heads were inoculated per replicate and 3 replicates were done per treatment; inoculated spikelets and rachis were harvested separately at 3 d after inoculation (dai). For Wuhan 1 and Nyubai, inoculated spikelet samples were collected in triplicate (from 5 heads per replicate) at 2 and 4 dai. For the DH population, only one replicate, composed of 5 to 6 whole heads, was done per line; it was harvested at 2 dai with *F. graminearum* (no water treatment was performed).

### RNA extraction and cDNA synthesis

For RT-qPCR analyses, total RNA was extracted using the TRI-Reagent (Molecular Research Center Inc) following manufacturer’s instructions, except for the following modification: the aqueous phase separation was technically implemented with phase-lock gel tubes (5 PRIME Inc., Gaithersburg, MD, USA) before the isopropanol precipitation. Crude total RNAs were cleaned up using the RNeasy Mini Kit (Qiagen, Mississauga, Canada), including a DNase I treatment from RNase-free DNase set (Qiagen), according to manufacturer’s instructions. RNA integrity and quality were initially tested by separation on denaturing 1% formaldehyde agarose gel electrophoresis; quantification and additional quality evaluation were performed using the QIAxpert instrument (Qiagen, Missisauga, Canada).

The cDNA synthesis of all RNA samples was carried out with the RETROscript® reverse transcription kit (Ambion), using 3 μg of each RNA sample into a 20 μl reaction volume with oligo(dT)18 primer, and all manipulations followed the manufacturer’s protocol.

### Data extraction from RNA-Seq datasets and database cross-referencing

**Fig. 1:**
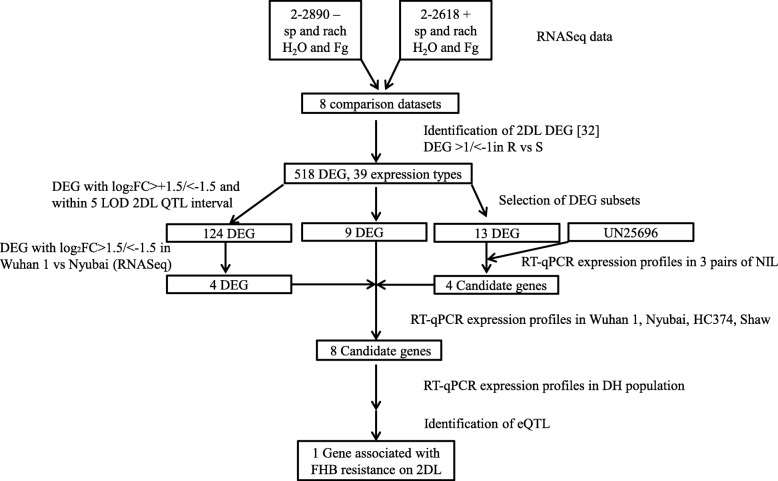
Overall strategy used in this study for the identification of candidate genes associated with the 2DL QTL for FHB resistance. The identification of DEG between tissues of a wheat NIL containing the 2DL QTL (2–2618) and a NIL without the 2DL QTL (2–2890) were described in Biselli et al. [32]. The RNA-Seq data for Wuhan 1 and Nyubai is described in [37]. *UN25696* was first identified as TA.25696 in [31]. sp., spikelet; rach, rachis; Fg, *F. graminearum*; DEG, differentially expressed genes; log_2_ FC, log base 2 fold change

The complete list of DEG [32] from an RNA-Seq dataset comparing the expression profiles of spikelet and rachis tissues from wheat heads of the NIL 2–2890 and 2–2618, respectively carrying the S (−) and R (+) allele for the 2DL QTL and sampled 3 days after treatment with *F. graminearum* or water, was re-examined (Fig. 1). The DEG from the chromosome arm 2DL were extracted, then those with a log_2_ fold change (FC) > 1.0/<− 1.0 for the 4 comparisons between 2 and 2890 and 2–2618 (R vs S_infected spikelet; R vs S_infected rachis; R vs S_mock spikelet; R vs S_mock rachis) were selected. The selected DEG were classified into expression patterns using each of the four R vs S comparisons and in either or both tissues for the Fg vs H_2_O S and R comparisons.

In addition, the 2DL DEG with a log_2_ FC > 1.5/<− 1.5 were BLAST-aligned to Wheat Genome IWGSC RefSeq v1.0 [38] to identify those located within the interval defined by the genetic markers Ku_c19185_1569 and cfd233. Expression ratios for those DEG in spikelet tissues from Wuhan 1 and Nyubai at 2 dai with *F. graminearum* treatment were extracted from an additional RNA-Seq dataset [37] for the selected DEG; log_2_ FC < 1.0/> − 1.0 were considered not significant.

### Bioinformatics analysis

The function BLASTp in NCBI [39] was used to identify homology to non-redundant protein sequences in *Arabidopsis*. Protein sequence alignments were done using either DNAMAN v8.0 (Lynnon Biosoft Corporation) or CLUSTAL multiple sequence alignment by MUSCLE (3.8) [40].

### Reverse transcription quantitative PCR (RT-qPCR)

For RT-qPCR analysis, three wheat genes, glyceraldehyde-3-phosphate dehydrogenase (GAPDH, *Traes_7AL_D93FC054C*), indole-3 acetaldehyde oxidase (IAAOx, *Traes_2AL_2C4546D2D*) and heterogeneous nuclear ribonucleoprotein Q (hn-RNPQ, *Traes_2AL_45601830C*) were used as the reference genes to normalize the expression data in the three pairs of NIL and in Wuhan 1, Nyubai; a fourth reference gene, amine oxidase (AOx, *Traes_2AL_CD28AB70E*) was used for normalisation with the DH population. Primers (Additional File 2) were designed using Integrated DNA Technologies (IDT) [41] and synthesized by Sigma Genosys Canada (Oakville, Ontario, Canada). The cDNAs were diluted for 30 times, and 5 μl of each 30 × −diluted cDNA were added into 25-μl reaction volumes using the SensiFast SYBR No-Rox kit (Bioline, London, UK). The RT-qPCR was carried out in a MJ Research PTC200 thermal Cycler with Chromo 4 detector with 10 min at 95 °C, 35 cycles of 30 s at 95 °C, 30 s at melting temperature (Additional File 2), and 1 min at 72 °C, melting curve from 55 °C to 95 °C, read every 1 °C, hold 5 s. For each pair of NIL, and for Wuhan 1 and Nyubai, two technical replicates and three biological replicates of each treatment were carried out. For the samples from the DH lines, only technical replicates were performed. The 2^-ΔΔCt^ method [42] was used to calculate the FC of the cycle threshold (Ct) value, and the relative expression levels were normalized against the three or four wheat reference genes as calculated by [43], and rescaled using the lowest value among compared samples for a given gene as 1.

### QTL analysis

A genetic map was constructed based on a 90 K Infinium SNP beadchip array [44] analysis of 104 lines from the Wuhan 1/Nyubai DH population and the SSR genotyping data formerly gathered by Somers et al. [20] (Additional File 3, columns A to C). Linkage analysis was conducted with MapDisto v. 1.7.7 [45]. A minimum LOD (logarithm of odds) score of 3.0 and a maximum recombination fraction of 0.3 were used to identify linkage groups. Recombination fractions were converted to map distances with the Kosambi mapping function. A single marker with the least missing data was retained from each linkage bin for QTL analysis of FHB phenotype data, which was conducted with QGene 4.3.10 [46]. Composite interval mapping determined the location of the 2DL QTL. The QTL analysis model excluded co-factors from chromosome 2D, so that calculation of QTL statistics would not be affected by a co-factor from this chromosome. Permutation analysis, based on 10,000 iterations, calculated the 5% LOD significance threshold to be 8.73. RT-qPCR data from 85 of the lines from the DH population were used for eQTL mapping; those lines generate a similar genetic interval as the original set for the 2DL QTL for FHB resistance. The relative expression value of a gene in each line was treated as phenotypic data. The location of eQTL was determined with simple interval mapping in QGene. The 10,000 permutation 5% LOD significance threshold was determined for the expression data of each candidate gene.

The genetic position of SNP markers in the consensus map of Wang et al. [44] as well as their physical position in IWGSC RefSeq v1.0, based on best BLAST alignments [38], is provided in Additional File 3, columns D to F and G to J respectively. Position of key SSR markers for the 2DL QTL for FHB resistance in IWGSC RefSeq v1.0 is also provided in Additional File 3. Position of best BLAST alignment in IWGSC RefSeq v1.0 [38] for the genes characterized in this study is provided in Table 1.

**Table 1:** 2DL DEG between R and S NIL that were further characterized by RT-qPCR

2DL DEG Name (IWGSC release 2.25)	Annotation	IWGSC RefSeq v1.0^e^			Expression pattern	RT-qPCR characterization		
		Name	Start	End		In 3 pairs of NIL	In Wuhan 1, Nyubai	In DH population
Traes_2DL_03CAA3B80^a^	copper-transporting ATPase PAA2, chloroplastic	TraesCS2D01G495500LC	493,192,302	493,195,914	2	yes	yes	yes
Traes_2DL_0A96AAC5B^a^	Sodium transporter protein	TraesCS2D02G428300	540,161,984	540,166,078	24	yes		
Traes_2DL_0AB0ABFD5	Malate synthase	TraesCS2D02G344200	440,325,740	440,328,360	27	yes		
Traes_2DL_179570792^a^	WD-40 repeat family protein	TraesCS2D02G440500	550,630,373	550,636,721	1	yes	yes	yes
Traes_2DL_3BBD24259^a^	Smr domain-containing protein	TraesCS2D02G415100	529,007,465	529,012,316	12	yes		
Traes_2DL_3F691BC13	Receptor kinase	TraesCS2D02G599300	650,752,287	650,757,581	9	yes		
Traes_2DL_44552FCF2	DnaJ / Sec63 Brl domains-containing protein	TraesCS2D02G275600	345,248,090	345,248,281	23	yes		
Traes_2DL_7B0056729	Serine incorporator	TraesCS2D02G347800	445,890,982	445,897,188	20	yes		
Traes_2DL_848E9C4A6^a^	Heat shock transcription factor	TraesCS2D02G399000	512,041,712	512,043,335	36	yes		
Traes_2DL_89A313AC3	Glycin-rich RNA-binding protein	TraesCS2D02G302400	385,568,830	385,570,145	28	yes	yes	
Traes_2DL_BB6F31152	Receptor protein kinase	TraesCS2D02G248200	291,952,536	291,956,775	9	yes		
Traes_2DL_E301DD433^a^	BTB/POZ and TAZ domain-containing protein 2	TraesCS2D02G339900	434,138,340	434,140,330	31	yes		
Traes_2DL_F784FBAA3^a^	2-oxoglutarate (2OG) and Fe(II)-dependent oxygenase	TraesCS2D02G392900	500,791,603	500,793,692	24	yes		
Traes_2DL_07F08C844^a,b^	cysteine-rich/transmembrane domain A-like protein	TraesCS2D02G417400	531,711,723	531,712,483	23		yes	yes
Traes_2DL_37D967CFC^a,b^	CC-NBS-LRR protein	TraesCS2D02G380500	484,858,484	484,862,886	23		yes	yes
Traes_2DL_382370E3B	Pectinesterase	TraesCS2D02G322500	413,777,752	413,779,866	18		yes	yes
Traes_2DL_5642F7EAC	Receptor-like protein kinase	TraesCS2D02G263100	320,280,150	320,283,723	10		yes	
Traes_2DL_647B61E84	1,4-alpha-glucan-branching enzyme	TraesCS2D02G290800	372,924,177	372,935,106	7		yes	
Traes_2DL_892F83E0B	Sec14p-like phosphatidylinositol transfer protein	TraesCS2D02G335700	429,053,162	429,057,812	18		yes	
Traes_2DL_8A90A6C4F^a^	Ankyrin repeat protein	TraesCS2D02G395500	506,771,094	506,784,989	39		yes	
Traes_2DL_8F75414FD	Protein kinase	TraesCS2D02G301200	383,845,349	383,849,177	9		yes	
Traes_2DL_A208876FE	Elongation factor 1-alpha	TraesCS2D02G320200	411,416,487	411,417,559	5		yes	yes
Traes_2DL_B7ABC1CB9^a,b^	Hypoxia-responsive protein	TraesCS2D02G438000	547,762,760	547,764,965	23		yes	yes
Traes_2DL_BA1B746DF	MLP protein	TraesCS2D02G328200	421,480,422	421,481,366	9		yes	
Traes_2DL_EDAEA1357^a,b^	Acidic endochitinase	TraesCS2D02G450600	560,399,016	560,400,258	31		yes^g^	
Traes_2DL_FA7A15F63	Glycerophosphodiester phosphodiesterase	TraesCS2D02G332500	425,740,309	425,746,371	10		yes	
UN25696^c,d^	Uncharacterized protein	(No gene model)^f^	446,264,922	446,266,534		yes	yes	yes
TaWRKY^c^ Traes_2DL_B8483F711	WRKY transcription factor	TraesCS2D02G489700	588,676,152	588,677,693			yes	yes
TaACT^c,d^	Agmatine coumaroyltransferase-2	TraesCS2D02G490400	589,282,163	589,283,855			yes	yes
NPR1-like^c^ Traes_2DL_14C8A084C	Receptor-like protein kinase	TraesCS2D02G572000	638,143,208	638,148,768			yes	yes

^a^DEG physically located in 5 LOD interval for FHB resistance 2DL QTL, based on IWGSC RefSeq v1.0 [38, 47]

^b^DEG with log_2_ FC > 1.5/<−1.5 for Wuhan 1/Nyubai at 2dai with *F. graminearum*

^c^Genes proposed to contribute to the 2DL QTL for FHB resistance in literature [31, 33, 48]

^d^Not annotated in Wheat Genome IWGSC release 2.25

^e^Best BLAST hit in IWGSC RefSeq v1.0 [38, 47]

^f^ Not annotated in Wheat Genome IWGSC RefSeq v1.0 [38, 47]

^g^No expression was detected by RT-qPCR

## Results

### Strategies to identify differentially expressed genes correlating with FHB resistance on the chromosome arm 2DL

In a previous analysis, the global expression profile changes in response to *F. graminearum* were described between two NIL carrying respectively the R and S allele for the FHB resistance QTL on chromosome arm 2DL [32]. That analysis used RNA-Seq to compare total mRNAs from spikelet and rachis tissues of the two NIL, 2–2890 and 2–2618, at 3 dai with either *F. graminearum* or water (mock treatment). The resulting list of DEG has been further investigated here, with a focus on the DEG associated with the 2DL chromosome arm; Fig. 1 illustrates the strategies used for the identification of candidate genes for the 2DL QTL for FHB resistance. The diversity of expression patterns among the DEG on 2DL was re-examined to identify DEG between the R and S NIL, either after the *F. graminearum* or the water treatment. Those DEG were further categorised using their response to the *F. graminearum* treatment. Of the 1406 DEG from the chromosome arm 2DL identified by Biselli et al. [32], 518 had a differential expression pattern between the R and S NIL and were regrouped into 39 expression patterns. A summary of the expression patterns observed is provided in Additional File 4, tab “expression patterns”. The most frequently observed expression pattern included DEG expressed at higher level in the spikelets of the R NIL than the S NIL, after one or both treatments, while being downregulated by the *F. graminearum* treatment (expression patterns 9 and 10); the other frequently observed expression pattern was that of DEG expressed at a lower level in the R NIL in one or both tissues while being upregulated by the *F. graminearum* treatment (expression patterns 23, 29 and 31). Expression ratios (log_2_ FC) for the 2DL DEG between R and S NIL, organised by expression patterns, are also provided in Additional File 4.

When the wheat reference sequence in Chinese Spring, IWGSC RefSeq v1.0 [38], became available, it was used to develop a list of 124 DEG with a log_2_ FC > 1.5/<− 1.5 between R and S NIL that were located between genetic markers Ku_c19185_1569 and cfd233 in IWGSC RefSeq v1.0 [38] (Additional File 3, Additional File 5). Those markers bracket a genomic region including the 2DL QTL and its five LOD drop support interval [20]. The genomic region was purposely selected to be larger than the 2 LOD drop interval commonly used to support the QTL. The expression profile of the 124 DEG was characterized in Wuhan 1 and Nyubai, the parents of the population where the 2DL QTL was originally identified, using RNA-Seq data that became available part way through this study [37]. The majority of the DEG (109/124) did not have a differential expression between Wuhan 1 and Nyubai.

The four DEG with a log_2_ FC > 1.5/<− 1.5 between Wuhan 1 and Nyubai at 2 dai with *F. graminearum* were selected for further expression studies (Table 1, Additional File 5). An additional subset of 22 DEG was also selected (Table 1, Additional File 4). Eight of these 22 DEG were also located within the 2DL-QTL genomic region, while the remaining 14 DEGs were selected because they encoded functions or expression profiles compatible with resistance to FHB, even though they were located outside of the 2DL QTL interval.

### Characterization of DEG expression profiles in additional material contrasting for the presence/absence of the 2DL QTL for FHB resistance

**Fig. 2:**
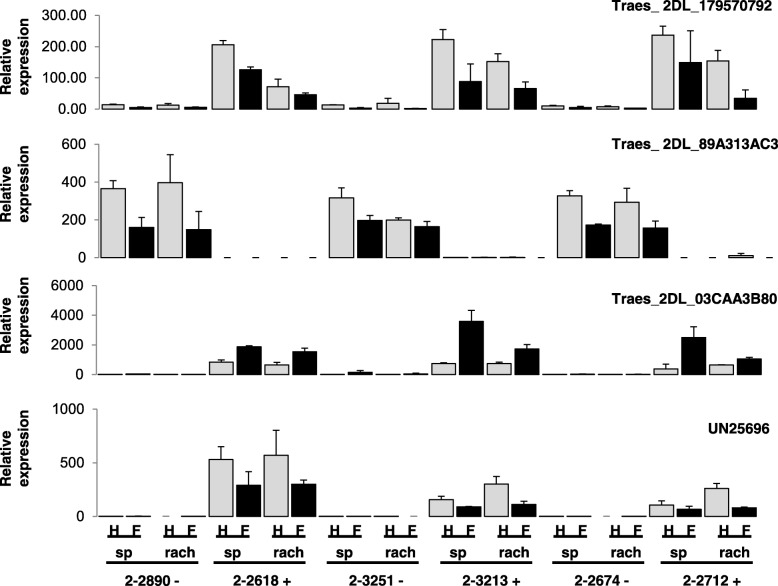
Relative expression patterns for three DEG and *UN25696* in three pairs of NIL segregating for the FHB resistance QTL on 2DL. RT-qPCR was carried out on three pairs of NIL using three biological replicates per treatment per NIL. *UN25696* was from [31]. H, water (mock) inoculation; F, *F. graminearum* inoculation; sp., spikelet; rach, rachis; − and +, S and R allele for 2DL QTL; no visible bar in a panel means that there was no detectable expression or expression level was too low to show on the figure

A group of thirteen 2DL DEG between R and S NIL [32], representing different expression patterns and a range of differential expression values, were selected for further characterization of their expression profile in additional NIL (Table 1, Fig. 1). RT-qPCR analysis was performed using water- and *F. graminearum*-treated spikelet and rachis tissues from 3 pairs of NIL, 2–2890 and 2–2618, 2–3251 and 2–3213, and 2–2674 and 2–2712, containing respectively the S and R alleles of the 2DL QTL in each pair (lineage provided in Additional File 1). Although all DEG had a consistent pattern of expression between the RNA-Seq data and the RT-qPCR analysis for the NIL 2–2890 and 2–2618, validating the RNA-Seq data, only three of the DEG tested showed a profile of differential expression that was consistent between the 3 pairs of NIL: *Traes_2DL_179570792*, *Traes_2DL_89A313AC3* and *Traes_2DL_03CAA3B80* (Fig. 2). *Traes_2DL_179570792* and *Traes_2DL_03CAA3B80* were expressed at a higher level in spikelet and rachis tissues of the three NIL carrying the R allele of the 2DL QTL while *Traes_2DL_89A313AC3* had a contrasting pattern of expression, showing higher expression in tissues of the NIL carrying the S allele. The ten other genes tested did not show a consistent expression profile between the three pairs of NIL (Additional File 6, data not shown).

*UN25696* (formerly called Ta.25696.1), a DEG previously identified in a microarray study as correlating with the 2DL QTL [31], was also tested in the three pairs of NIL (Table 1). It showed a consistent differential expression profile between the 3 pairs (Fig. 2). Expression of *UN25696* was very strongly upregulated in the NIL carrying the R allele of the 2DL QTL. Although it is not annotated in the gene models of the Wheat Genome IWGSC release 2.25, this DEG is located on chromosome 2DL (Table 1). Of the four genes showing a consistent expression profile between the three pairs of NIL, only *Traes_2DL_03CAA3B80* showed a significant increase in expression after infection, both in rachis and spikelets, while the other three were down-regulated by *F. graminearum* inoculation in one or both tissues (Fig. 2).

**Fig. 3:**
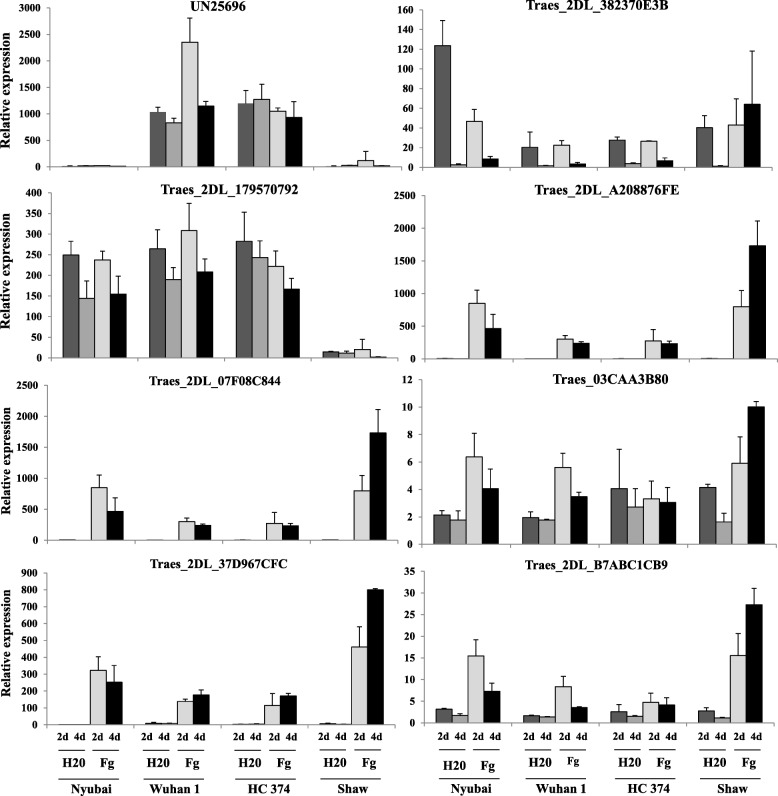
Relative expression patterns for seven DEG and *UN25696* in four genotypes. Wuhan 1 and HC374 carry the + allele for the FHB resistance QTL on 2DL, while Nyubai and Shaw carry the - allele. RT-qPCR was carried out using three biological replicates per treatment; samples were collected at 2 and 4 dai after water (H_2_O) or *F. graminearum* (Fg) inoculation

At this stage, four candidate genes were selected as having a consistent differential expression profile between the 3 pairs of NILs (*Traes_2DL_179570792*, *Traes_2DL_89A313AC3*, *Traes_2DL_03CAA3B80* and *UN25696*). The expression profile of these and thirteen of the additional selected DEG (Table 1, Additional File 4, Additional File 5) were characterized using spikelet samples collected at 2 and 4 dai with *F. graminearum* and water in four genotypes: Wuhan 1 and HC374, carrying the R allele for the 2DL QTL, and Nyubai and Shaw, carrying the S allele (Fig. 3). For *UN25696,* the results confirmed the strong differential expression between the genotypes carrying the R and S alleles for the 2DL QTL, as observed with the 3 pairs of NIL. Differential expression was consistently observed between the genotypes carrying the R and S alleles for *Traes_2DL_382370E3B*, *Traes_2DL_A208876FE*, *Traes_2DL_07F08C844*, *Traes_2DL_37D967CFC* and *Traes_2DL_B7ABC1CB9* (Fig. 3). However, some differences in expression of those genes were observed between Nyubai and Shaw, especially at 4 dai with *F. graminearum*; this may be explained at least in part by the difference in FHB response between Nyubai (which carries FHB QTLs on 3BS and 5AS) and Shaw (which does not carry any known FHB resistance QTL). A more modest expression difference was observed between the genotypes carrying the R and S alleles for *Traes_2DL_179570792* and *Traes_2DL_03CAA3B80*. Of those eight candidate genes, five were physically located in or near the 2DL QTL interval for FHB resistance. Eight other genes did not show consistent differences of expression between the genotypes carrying the R and S alleles for the 2DL QTL, including two which were located in or near that interval (Additional File 7, Table 1). No expression was detectable by RT-qPCR for *Traes_2DL_EDAEA1357*.

### Identification of candidate genes potentially contributing to the FHB resistance QTL on chromosome arm 2DL

To determine more precisely the level of association of the eight candidate genes with the 2DL QTL, their expression profile was determined in 85 DH lines derived from the cross Wuhan 1/Nyubai [20] (Table 1). Those lines were part of the mapping population used to identify the 2DL QTL. Expression profiles obtained by RT-qPCR for whole heads at 2 dai with *F. graminearum* are presented in Additional File 8. When compared with the level of FHB symptoms following single floret inoculation (Additional File 9), a modest trend towards increased expression of all candidate genes, except *Traes_2DL_37D967CFC*, was observed in lines with reduced FHB symptoms, with *Traes_2DL_03CAA3B80*, *Traes_2DL_382370E3B* and *Traes_2DL_A208876FE* showing the largest correlation (Additional File 10). It is interesting to note that there is a correlation of 1 between the expression profiles of *Traes_2DL_03CAA3B80* and *Traes_2DL_382370E3B* in the DH population, although those two genes have very different predicted functions and genomic positions (Table 1).

**Fig. 4:**
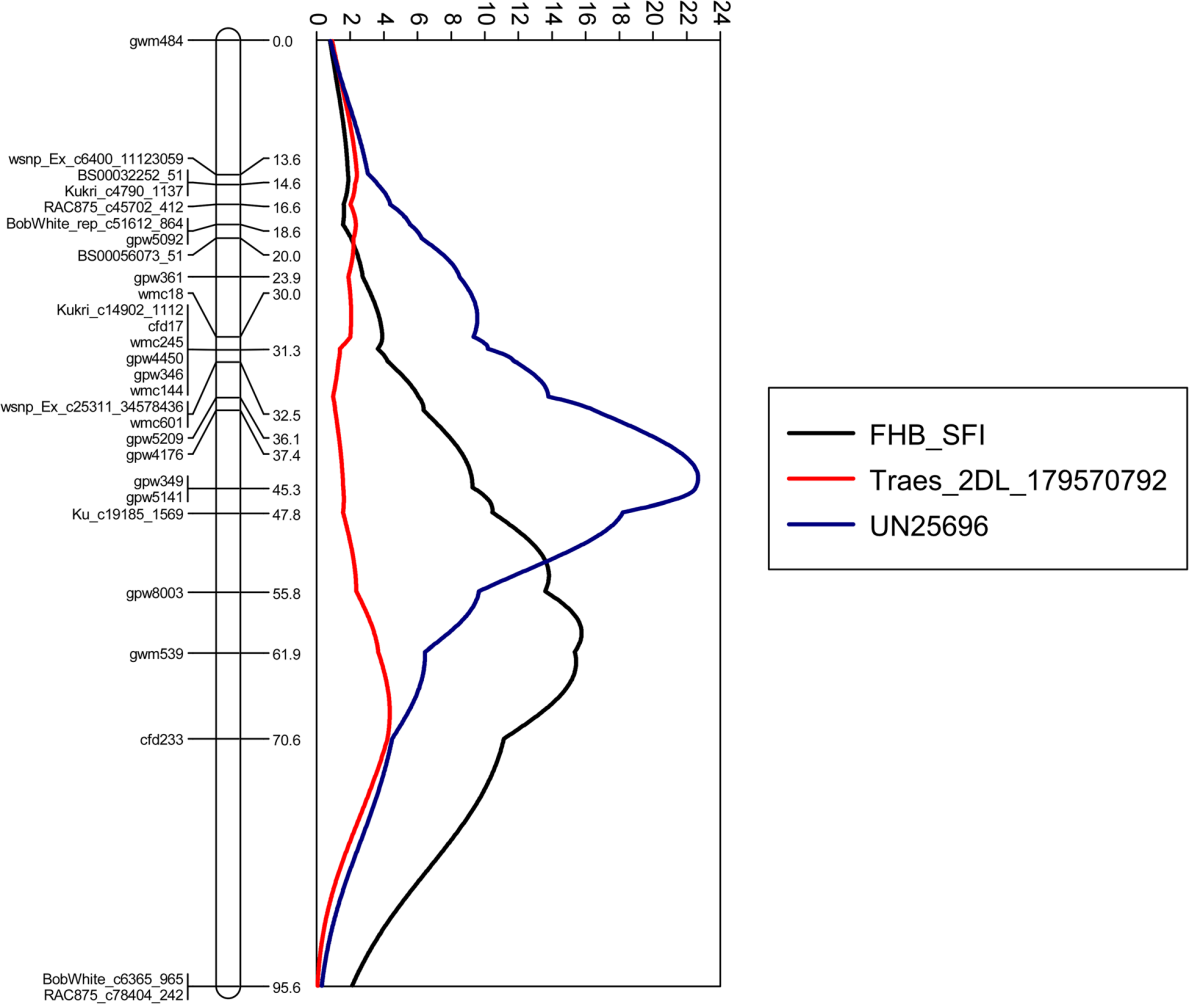
Genetic positioning of the FHB_SFI QTL and expression QTL for candidate genes on 2D genetic linkage map. The genetic linkage map of chromosome 2D was constructed from a wheat DH population of 104 lines while expression data from 85 lines were used for the eQTL mapping.

A map containing the original data from phenotyping and SSR genotyping for the Wuhan 1/Nyubai DH population [20] was supplemented with genotyping data from the wheat 90 K Infinium SNP beadchip array. The 2DL QTL for FHB resistance associated with single floret inoculation was mapped between markers gpw8003 and gwm539 using a two LOD drop support interval. The LOD peak for the FHB resistance QTL (position 59.9 cM on linkage group 2D.2) was close to gwm539 (position 61.9 cM, Table 2, Fig. 4). The relative expression values obtained for each gene with samples from the population were regarded as phenotypic data to identify eQTL for each gene. Two eQTL were detected on chromosome arm 2DL (Table 2) in the vicinity of the FHB_SFI QTL. The LOD peak for eQTL detected for *Traes_2DL_179570792* expression was at 67.9 cM on linkage group 2D.2, near cfd233, with a two LOD drop support interval between Ku_c19185_1569 and BobWhite_c6365_965. That portion of the linkage map is poorly defined because it has few markers. These results place this eQTL 8 cM from the LOD peak of the 2DL FHB resistance QTL. The LOD peak for the *UN25696* eQTL (position 42.2 cM) was 17.7 cM from the LOD peak for the 2DL FHB resistance QTL (Table 2**,** Fig. 4). No significant eQTL (using a 5% LOD threshold) were detected on 2DL for the other five candidate genes (Additional File 11). Interestingly, *Traes_2DL_07F08C844* and *Traes_2DL_37D967CFC* showed an eQTL above the 5% threshold on chromosome 5A, in the same genetic interval as the QTL for the FHB resistance gene *Fhb5* (Additional File 11).

**Table 2:** Characteristics of the FHB_SFI QTL and eQTL mapped to the chromosome arm 2DL

TraitName	Position (cM)	Support Interval (cM)^a^	LeftMarker	Right Marker	LOD	PVE (%)	Add	LOD Significance Threshold (5%)^b^
FHB_SFI^c^	59.9	55.9–67.4	gpw8003	gwm539	15.99	55.5	−7.86	8.73
UN25696	42.2	40.6–46.8	gpw4176	gpw5141	22.70	73.3	349	3.48
Traes_2DL_179570792	67.9	54.9–79.4	gwm539	cfd233	4.35	22.4	0.695	3.22
TaWRKY	125.9	–	gwm349	D_GDS7LZN02JJZ19_328	2.64	14.3	−0.616	3.37
TaACT	147.4	–	Excalibur_rep_c67599_2154	–	2.32	12.6	2.41	3.04
NPR1-like	155.7	–	BS00083623_51	–	2.21	12.1	−3.17	3.30

^a^Based on 2 LOD drop

^b^Based on 10,000 permutations

^c^Percent infected florets per spike at 21 days after single floret inoculation with *Fusarium graminearum* in a greenhouse environment, as described in Somers et al. [20]

### Characterization of candidate genes for the FHB resistance associated with 2DL from literature

**Fig. 5:**
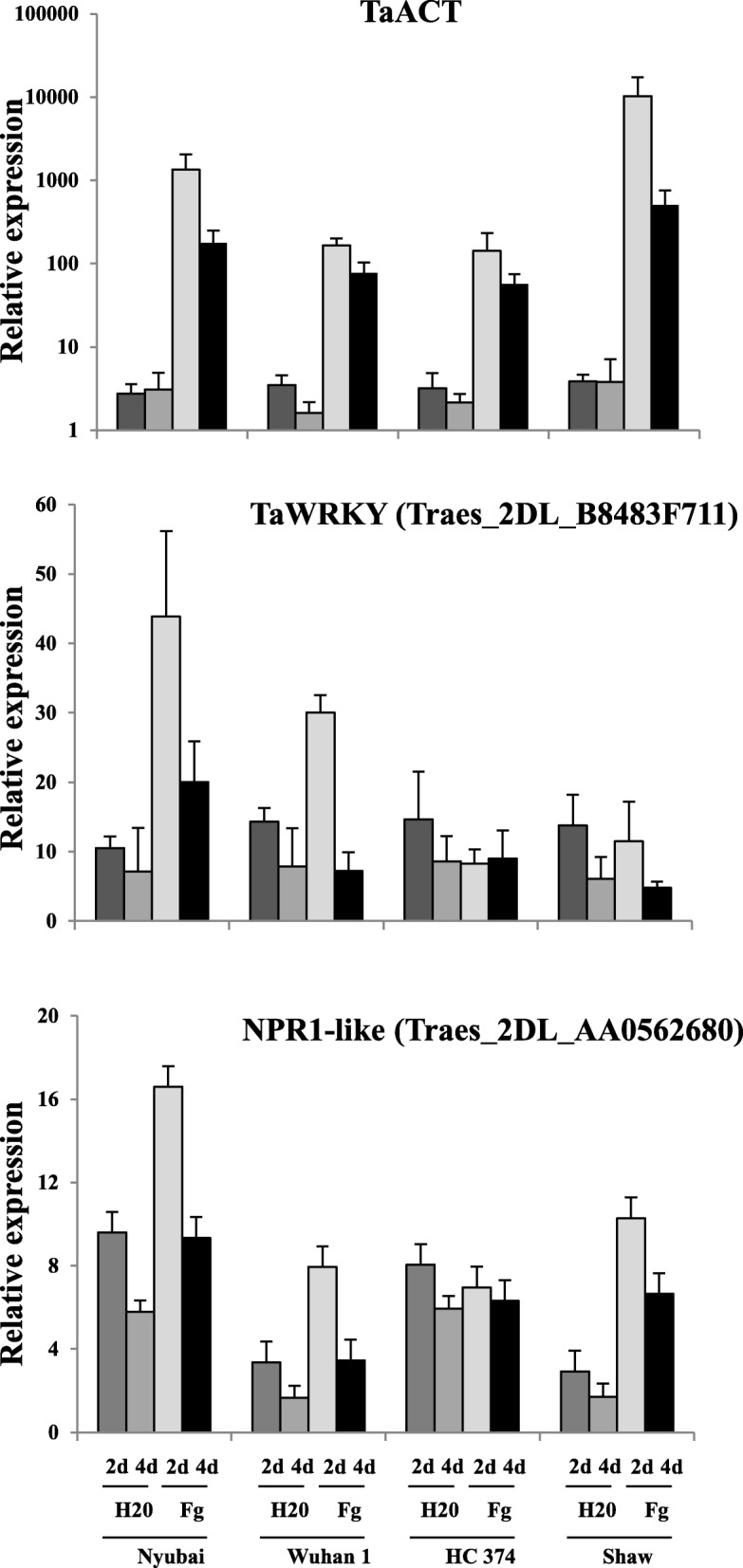
Relative expression patterns for *TaWRKY70*, *TaACT* and *NPR1*-like. Wuhan 1 and HC374 carry the + allele for the FHB resistance QTL on 2DL, while Nyubai and Shaw carry the - allele. RT-qPCR was carried out using three biological replicates per treatment; samples were collected at 2 and 4 dai after water (H_2_O) or *F. graminearum* (Fg) inoculation. *TaWRKY70*, *TaACT* and *NPR1*-like were identified in [33, 48]

Additional genes located on 2DL that have recently been proposed to contribute to the 2DL QTL [33] or to FHB resistance in general [48] were also characterized: *TaWRKY* (*Traes_2DL_B8483F711*), *TaACT* (not found in Wheat Genome IWGSC release 2.25), *TaDGK* (*Traes_2DL_9E3786CCA)*, *TaGLI1* (*Traes_2DL_ABA700EBD*) and *NPR1*-like (*Traes_2DL_14C8A084C*). Using RT-qPCR, *TaACT* showed significant differences in expression between genotypes carrying the R and S alleles of the 2DL QTL; such consistent differences were not observed for *TaWRKY* and *NPR1*-like (Fig. 5). No difference was observed for *TaDGK* and *TaGLI1* (data not shown). Expression profiles were also determined in the DH lines for *TaWRKY*, *TaACT* and *NPR1*-like (Additional File 8); eQTL detected on chromosome 2D for those three genes were completely outside of the interval for the 2DL QTL and were below the 5% LOD threshold (Table 2, Additional File 11). The only significant eQTL identified was for *NPR1*-like and localized on chromosome 2A.

### Functional annotation of two candidate genes with eQTL in the vicinity of the interval for the 2DL QTL

Protein alignment between *Traes_2DL_179570792* and the homeologous genes from wheat genomes A and B showed multiple differences between the three genomes, including a unique additional 56 amino acids at the C-terminus of *Traes_2DL_179570792* (Additional File 12). BLAST alignment against publicly available sequence databases showed that *Traes_2DL_179570792* had 89% similarity to an uncharacterized protein in *T. urartu* and about 50% similarity to the LisH and CTLH motifs present in many WD-40 repeat protein from *Arabidopsis* The homology with the WD-40 proteins was between amino acid 34 and 93 of *Traes_2DL_179570792*, in a segment identic between genomes A and D however presenting a deletion in genome B.

*UN25696* did not have a homolog to any other known or predicted protein. The corresponding genomic sequence on 2DL revealed a perfect match to *UN25696*; in addition, the genomic sequences available suggested that the homeologous sequences to *UN25696* on 2AL and 2BL either coded for truncated proteins or were pseudogenes (Additional File 13). *UN25696* had small domains (250–300 bp) of homology to a putative S-adenosyl methionine (SAM) methyltransferase and to a receptor kinase. *UN25696* was not included in the gene models of the Wheat Genome IWGSC release 2.25 [49]

## Discussion

*Fusarium* head blight is a destructive wheat disease, since it can sharply decrease yield and contaminate grain with DON, and further cause harm in human and livestock through consumption. Numerous genetic and molecular experiments have been performed towards the understanding of the mechanisms of resistance to FHB, including resistance to initial infection (type I) and to spread within the spike (type II); genomic regions associated with resistance QTL have been detected on all wheat chromosomes [1]. One moderate resistance QTL for type II resistance has been mapped on the long arm of the chromosome 2D [20]. Long et al. [31] have also shown that the presence of the 2DL QTL in breeding lines decreased the spread of infection and the amount of fungal biomass and DON accumulated in *F. graminearum*-infected tissues in greenhouse experiments; disease symptoms were also reduced under field conditions.

A comparison of RNA-Seq expression profiles between a pair of NIL with or without the R allele for the 2DL FHB-resistance QTL identified more than 1400 DEG located on the 2DL chromosome arm [32]. Organising the DEG in expression patterns to focus on genes which expression change was associated with the presence of the 2DL QTL rather than with the response to infection reduced the number of DEG to 518; 26 of those DEG were selected for further characterization. Three of the characterized DEG, as well as the gene *UN25696* showed a consistent pattern of expression in the spikelet and rachis tissues of 3 pairs of NIL segregating for the 2DL QTL. *Traes_2DL_03CAA3B80*, *Traes_2DL_179570792* and *UN25696* and five additional DEG also showed a consistent difference in expression between two genotypes carrying the R allele for the FHB-resistance QTL on 2DL, Wuhan 1 and HC374, and two genotypes carrying the S allele, Nyubai and Shaw. Two of those eight genes were then shown to have an eQTL on 2DL that mapped in the vicinity of the 2DL FHB_SFI resistance QTL, with only *Traes_2DL_179570792* overlapping with the FHB resistance QTL based upon two LOD drop support intervals. Increased expression of the genes *UN25696* and *Traes_2DL_179570792* was associated with reduced infection in the DH lines. *Traes_2DL_179570792* is the first expression marker associated with the 2DL QTL for FHB resistance. It may contribute directly to the QTL activity; however, more characterization will be required to support that. Moreover, a contribution to the 2DL QTL activity from *UN25696* cannot be definitively excluded, yet it is less likely.

It is interesting to note that two other candidate genes, *Traes_2DL_07F08C844* and *Traes_2DL_37D967CFC*, showed an eQTL above the LOD threshold on chromosome 5A. The Wuhan 1/Nyubai DH population also segregates for a FHB resistance QTL on 5A, sometimes referred to as *Fhb5* [20, 50]. It is possible that those two genes contribute to the resistance associated with *Fhb5*. No candidate gene has been identified yet for *Fhb5* nor its mechanism of action been defined.

A group of genes including *TaWRKY*70, *TaACT*, *TaDGK* and *TaGLI1*, have recently been proposed as candidate genes for the 2DL FHB resistance QTL [33]. The authors have demonstrated that *TaWRKY70* regulates the other three genes and contributed to reduction of infection by FHB in wheat heads. Allelic variation in a *NPR1*-like gene, also located on the chromosome arm 2DL, has been shown to be associated with FHB resistance in wheat [48]. Although those five genes are located on the 2DL chromosome arm, their physical locations in IWGSC RefSeq v1.0 [38] were outside of the genetic interval for the 2DL QTL (Table 1, data not shown). In addition, our analysis showed that their eQTL on chromosome 2D in the DH population from Wuhan 1/Nyubai were not significant and outside of the 2DL QTL mapping interval. This strongly suggests that those genes are unlikely candidate genes for that QTL, even though they contribute to FHB resistance. *NPR1*-like had a significant eQTL on chromosome 2A; however, no QTL associated with FHB resistance has been identified on 2A in that population.

Functional annotation has shown that *Traes_2DL_179570792* has homology with the LisH and CTLH motifs present in WD-40 proteins. WD-40 proteins are part of a large family of proteins with roles in coordination of large protein complex assemblies and are involved in a broad range of biological functions [51]. Some WD-40 proteins have LisH and CTLH motifs in addition to the WD-40 repeats, as is the case for *Traes_2DL_179570792*; the LisH and CTLH motifs confers oligomerization properties to the proteins. Very few of those LisH/CTLH-containing WD-40 proteins have been functionally characterized in plants. These include the *Arabidopsis* protein TOPLESS, a corepressor which is key in the regulation of hormone signaling and development [52] and the *Arabidopsis* protein WDR26, which coordinates cellular response to light, stresses and hormone changes [53].

*UN25696* is likely an orphan gene, possibly explaining why it has not been annotated as a gene in the Wheat Genome IWGSC release 2.25 [49]. Small domains (250–300 bp) of homology to a putative S-adenosyl methionine (SAM) methyltransferase and to a receptor kinase in *UN25696*, raise the possibility that it is involved in cell signaling associated with plant defense. SAM-methyltransferases and receptor kinases are part of large gene families with roles in many biological processes. SAM-methyltransferases can play a role in gene regulation, metabolite synthesis and cell signaling [54]. For example, the *Arabidopsis thaliana* gene AtHOL1, which has SAM-methyltransferase activity and is part of the biosynthetic pathway for methylthiocyanate, contributed to defense against *Pseudomonas syringae* pv. Maculicola [55]. Receptor kinases are well known for their role in cell signaling, including for plant defense [56, 57].

## Conclusions

In previous reports, microarray and RNA-Seq analyses have been performed on a pair of NIL segregating for the 2DL QTL for FHB resistance. Among the DEG identified in those analyses as belonging to the chromosome arm 2DL, 26 were further characterized either in additional pairs of NIL segregating for the 2DL QTL and/or in two pairs of genotypes carrying either the S or R allele for that QTL. Eight candidate genes showed a consistent pattern of expression in the material tested. The expression profiles of those eight candidate genes were further characterized in 85 DH lines from a mapping population derived from the cross Wuhan 1/Nyubai, identifying two genes, *Traes_2DL_179570792* and *UN25696*, with an eQTL overlapping with or in the vicinity of the 2DL QTL. Additional experiments involving functional validation (eg. gene silencing or overexpression) will be required to determine if one or both of these genes contribute directly or indirectly to the FHB resistance associated with the 2DL QTL. *Traes_2DL_179570792* is the first expression marker associated with the 2DL QTL and is adding information to a poorly define area of the linkage map.

## Additional files

Additional file 1: Lineages of three pairs of NIL contrasting for the presence or absence of the 2DL QTL for FHB resistance. The far left column illustrates the crossing strategy for developing the NIL. The remaining three columns indicate respectively the lineages and names of the three pairs of NIL developed from two separate F2 plants. Seeds from the BC_2_F_4_ generation were used for our experiments. R and S, FHB-resistant and susceptible plant respectively; BC_n_F_n_, backcross generation n and self-cross generation n; +, NIL carrying only the R allele for the 2DL QTL; −, NIL carrying the S allele for the 2DL, 3BS and 5A QTL for FHB resistance. Modified from Long et al. [31]. (PPTX 67 kb)

Additional file 2: RT-qPCR primer pairs for wheat normalisation genes and for DEG further characterized. (XLSX 17 kb)

Additional file 3: Linkage map for the Wuhan 1/Nyubai DH population. Genetic position of SNP markers in Wang et al. [44] and position of SNP and key SSR markers in IWGSC RefSeq v1.0 are also provided. Chr, chromosome; Pos, genetic position in cM; score, Best BLAST alignment score in RefSeq v1.0. (XLSX 889 kb)

Additional file 4: Expression profile patterns for genes differentially expressed between R and S NIL and located on the 2DL chromosome arm. Expression profiles are expressed as log_2_ FC. Data for eight comparison pairs is provided; only log_2_ FC > 1.0/<− 1.0 are presented. RNA-Seq analyses and comparisons are described in [32]. Blue highlights indicate genes that were further characterized in three pairs of R/S NIL and/or Wuhan 1 and Nyubai (see Table 1). Positive and negative log_2_ FC values are highlighted in red and green, respectively. ID, gene model numbers in Wheat Genome IWGSC release 2.25 [49]; R, resistant genotype; S, susceptible genotype; rach, rachis; sp., spikelet; Fg, infection with *F. graminearum*; H_2_O, mock inoculation with water. (XLSX 109 kb)

Additional file 5: Expression ratio (log_2_ FC) between Wuhan 1 and Nyubai spikelets at 2 dai with *F. graminearum* for DEG located within a genomic region including the 2DL QTL and differentially expressed between R and S NIL. Expression values for Wuhan 1 and Nyubai were from RNA-Seq data [37]. The genomic region was defined by markers Ku_c19185_1569 and cfd233. DEG with log_2_ FC > 1.5/<− 1.5 between the R and S NIL, indicated with purple highlight, were selected for further analysis. All DEG located in that genomic region that were further characterized are indicated with blue highlight. (XLSX 16 kb)

Additional file 6: Examples of expression profiles obtained by RT-qPCR for additional DEG in three pairs of NIL contrasting for the presence or absence of the 2DL QTL for FHB resistance. Spikelets and rachis from heads treated with either water (H) or *F. graminearum* (F) and sampled at 3 dai were used. Relationship between NIL is presented in Additional File 1. (PPTX 2891 kb)

Additional file 7: Expression profiles for eight DEG in four genotypes carrying either the R (+) allele for the 2DL QTL, Wuhan 1 and HC374, or the S (−) allele for that QTL, Nyubai and Shaw. Expression profiles were obtained by RT-qPCR on RNA from inoculated spikelets. Inoculations were with *F. graminearum* (Fg) and mock inoculation with water (H_2_O); sampling was at 2 and 4 dai (2d and 4d). (PPTX 12115 kb)

Additional file 8: Expression profiles for eight candidate genes in 85 DH lines of the mapping population derived from Wuhan 1/Nyubai. Expression profiles were obtained by RT-qPCR on RNA from whole heads infected by *F. graminearum* and sampled at 2 dai. *TaWRKY70*, *TaACT* and *NPR1*-like were identified in [33, 48]. When known, presence (+) or absence (−) of the 2DL QTL for FHB resistance is indicated for each NIL and the 2 parents in a column to the right of the graphs, based on the presence of the Wuhan 1 or Nyubai allele for gwm539. (XLS 617 kb)

Additional file 9: FHB symptoms for 85 DH lines of the mapping population derived from Wuhan 1/Nyubai. Average percent infected florets per head after single floret inoculation, as described in Table 2. When known, presence (+) or absence (−) of the 2DL QTL for FHB resistance is indicated below the name of each NIL and the 2 parents, based on the presence of the Wuhan 1 or Nyubai allele for gwm539. (PPTX 71 kb)

Additional file 10: Correlation between FHB symptoms following single floret inoculation (FHB_SFI) and expression of candidate genes in the DH population derived from Wuhan 1/Nyubai. (DOCX 23 kb)

Additional file 11: Expression QTL (eQTL) detected using the RT-qPCR expression data in the DH population for 8 candidate genes and 3 additional genes from literature. For each gene, the chromosome(s) where an eQTL is detected, together with its 10,000 permutation LOD and 5% LOD significance threshold are provided. eQTL with LOD above the threshold are marked in green. *TaWRKY70*, *TaACT* and *NPR1*-like were identified in [33, 48]. (XLSX 12 kb)

Additional file 12: Amino acid sequence alignment for *Traes_2DL_179570792* and the homeologous genes on wheat genomes A and B. 2D, *Traes_2DL_179570792*; 2B, *Traes_2BL_410E9E91D*; 2A.1 to 2A.3, predicted protein isoforms of *Traes_2AL_2079C6E79*. Asterisks and dots under the aligned amino acids indicate homology between sequences. (PPTX 505 kb)

Additional file 13: Nucleotide (A) and amino acid (B) sequences alignment of the Unigene *UN25696* (represented in the NCBI EST collection by the accession CD373927) with homeologous genomic sequences from chromosomes 2AL, 2BL and 2DL (as in IWGSC release 2.25). The arrows define the predicted borders of the coding sequence; the single base InDels are indicated by a black triangle; the larger InDel sequence in the B genome is boxed; the asterisks indicate premature stop codons. (PPTX 141 kb)

## Abbreviations

Dai: Days after inoculation
DEG: Differentially expressed gene(s)
DH: Population/line: doubled haploid population/line
DON: Deoxynivalenol
eQTL: Expression quantitative trait locus
FC: Fold change
FHB: Fusarium head blight
FHB_SFI QTL: QTL for FHB resistance following single floret inoculation
NIL: Near isogenic line(s)
R or (+) allele: Resistant allele
RT-qPCR: Reverse transcription quantitative real-time polymerase chain reaction
S or (−) allele: Susceptible allele
SAM: S-Adenosylmethionine
SFI: Single floret inoculation
SSR: Simple sequence repeat
