# Correction to: Identification and mapping of expressed genes associated with the 2DL QTL for fusarium head blight resistance in the wheat line Wuhan 1

**DOI:** 10.1186/s12863-019-0777-1

**Published:** 2019-10-16

**Authors:** Xinkun Hu, Hélène Rocheleau, Curt McCartney, Chiara Biselli, Paolo Bagnaresi, Margaret Balcerzak, George Fedak, Zehong Yan, Giampiero Valè, Shahrokh Khanizadeh, Thérèse Ouellet

**Affiliations:** 1 Ottawa Research and Development Centre, Agriculture and Agri-Food Canada, 960 Carling Ave, Ottawa, ON K1A 0C6 Canada; 2 Triticeae Research Institute, Sichuan Agricultural University, 211 Huimin Road, Wenjiang, Chengdu, Sichuan 611130 People’s Republic of China; 3 Morden Research and Development Centre, Agriculture and Agri-Food Canada, 101 Route 100, Unit 100, Morden, Manitoba R6M 1Y5 Canada; 4 CREA, Council for Agricultural Research and Economics - Research Centre for Genomics and Bioinformatics, Via S. Protaso 302, I-29017 Fiorenzuola d’Arda, PC Italy; 5 Dipartimento di Scienze e Innovazione Tecnologica, Università del Piemonte Orientale, Vercelli, Italy

**Correction to: BMC Genet (2019) 20:47**

**https://doi.org/10.1186/s12863-019-0748-6**

Following publication of the original article [1], we have been notified that some important information was omitted by the authors in the Copyright note. The Copyright note should read as below.

© Her Majesty the Queen in Right of Canada as represented by the Minister of Agriculture and Agri-Food Canada, 2019.

The original article has been corrected.

## References

[1] Hu X (2019). Identification and mapping of expressed genes associated with the 2DL QTL for fusarium head blight resistance in the wheat line Wuhan 1. BMC Genet.

