# Supplementary material for: Identification and mapping of expressed genes associated with the 2DL QTL for fusarium head blight resistance in the wheat line Wuhan 1
Source: BMC Genet. 2019 May 21;20:47. doi: 10.1186/s12863-019-0748-6 (PMC6528218; doi:10.1186/s12863-019-0748-6)
Supplement: Supplementary file 1 — Lineages of three pairs of NIL contrasting for the presence or absence of the 2DL QTL for FHB resistance. The far left column illustrates the crossing strategy for developing the NIL. The remaining three columns indicate respectively the lineages and names of the three pairs of NIL developed from two separate F2 plants. Seeds from the BC2F4 generation were used for our experiments. R and S, FHB-resistant and susceptible plant respectively; BCnFn, backcross generation n and self-cross generation n; +, NIL carrying only the R allele for the 2DL QTL; −, NIL carrying the S allele for the 2DL, 3BS and 5A QTL for FHB resistance. Modified from Long et al. [31]. (PPTX 67 kb) [file 12863_2019_748_MOESM1_ESM.pptx]

## Slide 1
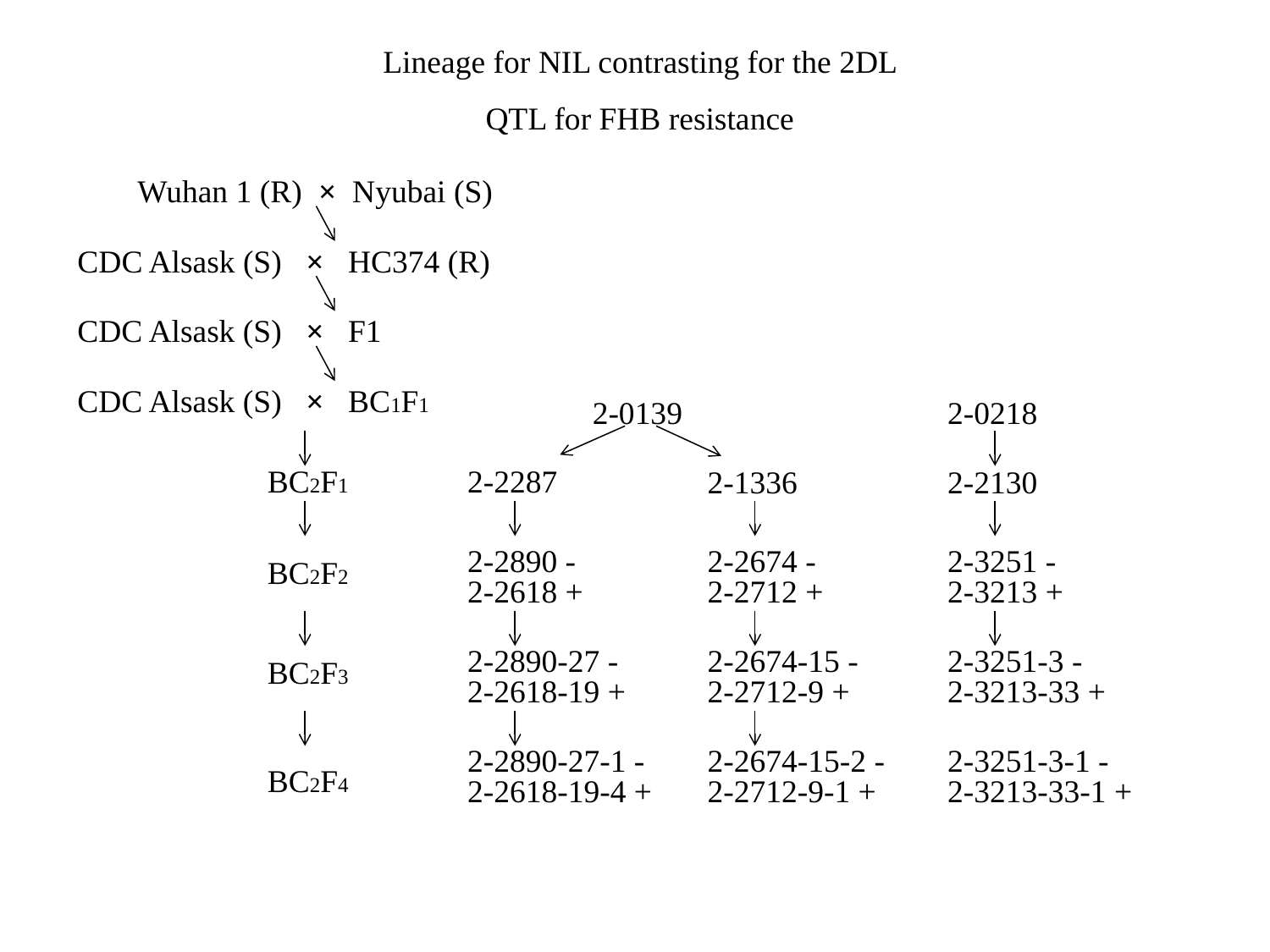

Lineage for NIL contrasting for the 2DL QTL for FHB resistance
Wuhan 1 (R) × Nyubai (S)
CDC Alsask (S) × HC374 (R)
CDC Alsask (S) × F1
CDC Alsask (S) × BC1F1
2-0139
2-0218
BC2F1
2-2287
2-1336
2-2130
2-2890 -
2-2674 -
2-3251 -
BC2F2
2-2618 +
2-2712 +
2-3213 +
2-2890-27 -
2-2674-15 -
2-3251-3 -
BC2F3
2-2618-19 +
2-2712-9 +
2-3213-33 +
2-2890-27-1 -
2-2674-15-2 -
2-3251-3-1 -
BC2F4
2-2618-19-4 +
2-2712-9-1 +
2-3213-33-1 +
