# Supplementary material for: Identification and mapping of expressed genes associated with the 2DL QTL for fusarium head blight resistance in the wheat line Wuhan 1
Source: BMC Genet. 2019 May 21;20:47. doi: 10.1186/s12863-019-0748-6 (PMC6528218; doi:10.1186/s12863-019-0748-6)
Supplement: Supplementary file 6 — Examples of expression profiles obtained by RT-qPCR for additional DEG in three pairs of NIL contrasting for the presence or absence of the 2DL QTL for FHB resistance. Spikelets and rachis from heads treated with either water (H) or F. graminearum (F) and sampled at 3 dai were used. Relationship between NIL is presented in Additional File 1. (PPTX 2891 kb) [file 12863_2019_748_MOESM6_ESM.pptx]

## Slide 1
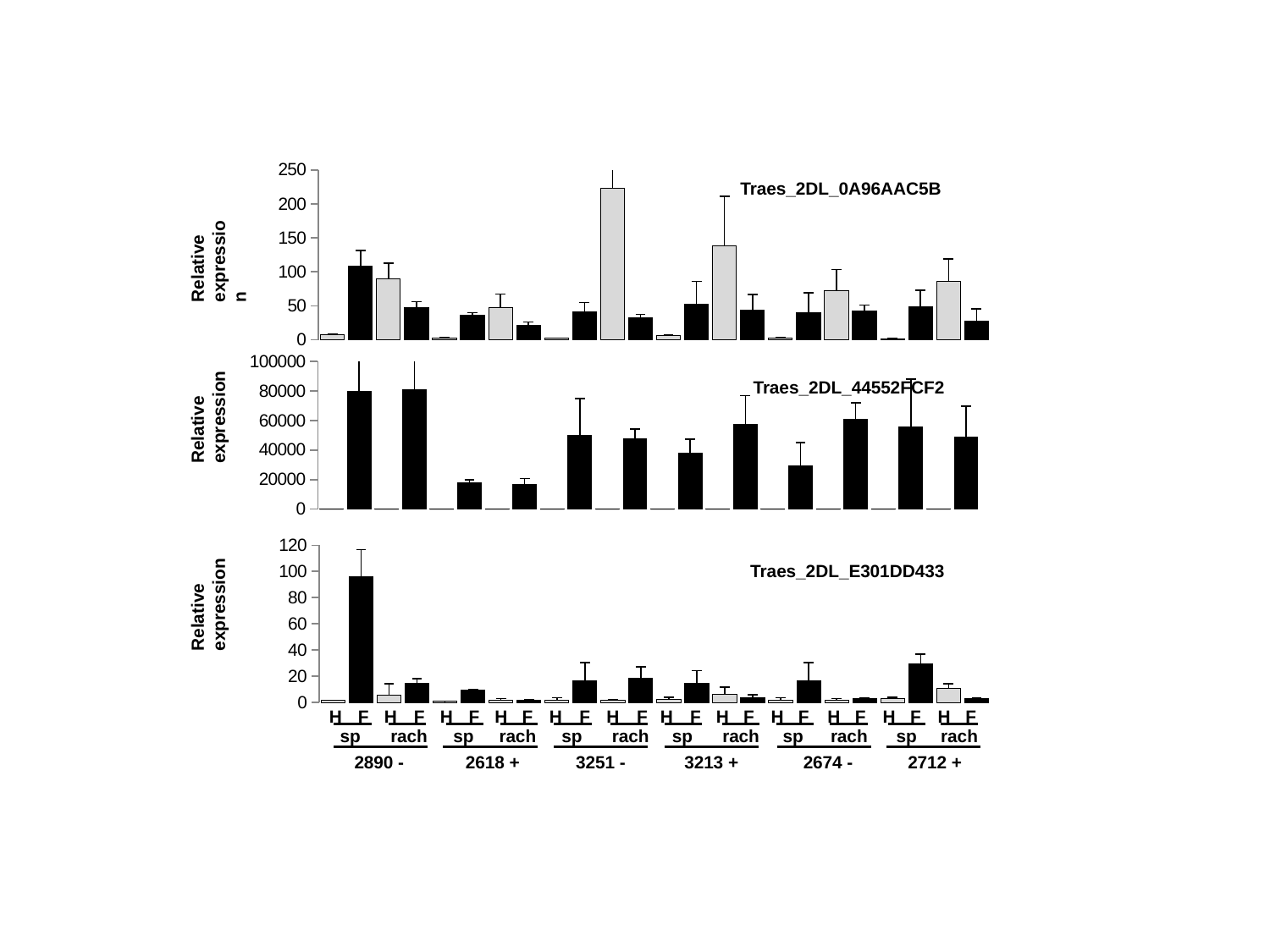

### Chart
| Category | |
|---|---|
| 2890 H20 sp | 8.072793405894805 |
| 2890 Fg sp | 108.46580169919092 |
| 2890 H20 ra | 89.49985129297272 |
| 2890 Fg ra | 47.813867458555244 |
| 2618 H20 sp | 2.6201806560102887 |
| 2618 Fg sp | 36.528674195284516 |
| 2618 H20 ra | 47.46858348598473 |
| 2618 Fg ra | 21.284804877757317 |
| 3251 H20 sp | 2.5973584728849253 |
| 3251 Fg sp | 40.8719424189381 |
| 3251 H20 ra | 222.84972174982258 |
| 3251 Fg ra | 32.75188617300886 |
| 3213 H20 sp | 5.797200303014223 |
| 3213 Fg sp | 52.955530593625575 |
| 3213 H20 ra | 138.8349363049468 |
| 3213 Fg ra | 43.73956858995452 |
| 2674 H20 sp | 2.1091448481256343 |
| 2674 Fg sp | 39.72487432802604 |
| 2674 H20 ra | 71.83135910171329 |
| 2674 Fg ra | 42.80879890494585 |
| 2712 H20 sp | 1.3759123884040099 |
| 2712 Fg sp | 49.02797733514088 |
| 2712 H20 ra | 86.1902073827875 |
| 2712 Fg ra | 27.158129824787824 | Traes_2DL_0A96AAC5B
Relative expression
### Chart
| Category | |
|---|---|
| 2890 H20 sp | 3.285308650940531 |
| 2890 Fg sp | 79743.4508507643 |
| 2890 H20 ra | 63.61050507439273 |
| 2890 Fg ra | 80716.48723531427 |
| 2618 H20 sp | 6.47548143579679 |
| 2618 Fg sp | 17935.8664911114 |
| 2618 H20 ra | 6.971920242056715 |
| 2618 Fg ra | 16924.026234419223 |
| 3251 H20 sp | 10.882054059907668 |
| 3251 Fg sp | 49909.33201842152 |
| 3251 H20 ra | 11.751707976308467 |
| 3251 Fg ra | 47932.329289867565 |
| 3213 H20 sp | 2.0563977965225106 |
| 3213 Fg sp | 38132.69691272063 |
| 3213 H20 ra | 6.0141559050587885 |
| 3213 Fg ra | 57538.71186132118 |
| 2674 H20 sp | 1.854072999164887 |
| 2674 Fg sp | 29356.58139733746 |
| 2674 H20 ra | 6.36589742897182 |
| 2674 Fg ra | 60981.28510063058 |
| 2712 H20 sp | 3.5228638893994266 |
| 2712 Fg sp | 55864.599073130055 |
| 2712 H20 ra | 6.05992133298967 |
| 2712 Fg ra | 48994.130495124555 |Relative expression
 Traes_2DL_44552FCF2
### Chart
| Category | |
|---|---|
| 2890 H20 sp | 1.5343749498025865 |
| 2890 Fg sp | 96.09493233251142 |
| 2890 H20 ra | 5.785481069932165 |
| 2890 Fg ra | 14.40982011378898 |
| 2618 H20 sp | 0.7196324413802123 |
| 2618 Fg sp | 9.118118880873725 |
| 2618 H20 ra | 1.4950520744283053 |
| 2618 Fg ra | 1.725437756099829 |
| 3251 H20 sp | 1.8072170162623629 |
| 3251 Fg sp | 16.525622949853638 |
| 3251 H20 ra | 1.4667599504690683 |
| 3251 Fg ra | 18.238901526186066 |
| 3213 H20 sp | 2.383410987803682 |
| 3213 Fg sp | 14.808973819769543 |
| 3213 H20 ra | 6.209613846812076 |
| 3213 Fg ra | 3.769976020645451 |
| 2674 H20 sp | 1.8072170162623629 |
| 2674 Fg sp | 16.525622949853638 |
| 2674 H20 ra | 1.6250399999011502 |
| 2674 Fg ra | 3.238037467470042 |
| 2712 H20 sp | 3.199691633810401 |
| 2712 Fg sp | 29.55394410197053 |
| 2712 H20 ra | 10.688369980554024 |
| 2712 Fg ra | 2.8660490618581083 |Relative expression
 Traes_2DL_E301DD433
H
F
H
F
H
F
H
F
H
F
H
F
H
F
H
F
H
F
H
F
H
F
H
F
sp
rach
sp
rach
sp
rach
sp
rach
sp
rach
sp
rach
2890 -
2618 +
3251 -
3213 +
2674 -
2712 +
