# Supplementary material for: Identification and mapping of expressed genes associated with the 2DL QTL for fusarium head blight resistance in the wheat line Wuhan 1
Source: BMC Genet. 2019 May 21;20:47. doi: 10.1186/s12863-019-0748-6 (PMC6528218; doi:10.1186/s12863-019-0748-6)
Supplement: Supplementary file 7 — Expression profiles for eight DEG in four genotypes carrying either the R (+) allele for the 2DL QTL, Wuhan 1 and HC374, or the S (−) allele for that QTL, Nyubai and Shaw. Expression profiles were obtained by RT-qPCR on RNA from inoculated spikelets. Inoculations were with F. graminearum (Fg) and mock inoculation with water (H2O); sampling was at 2 and 4 dai (2d and 4d). (PPTX 12115 kb) [file 12863_2019_748_MOESM7_ESM.pptx]

## Slide 1
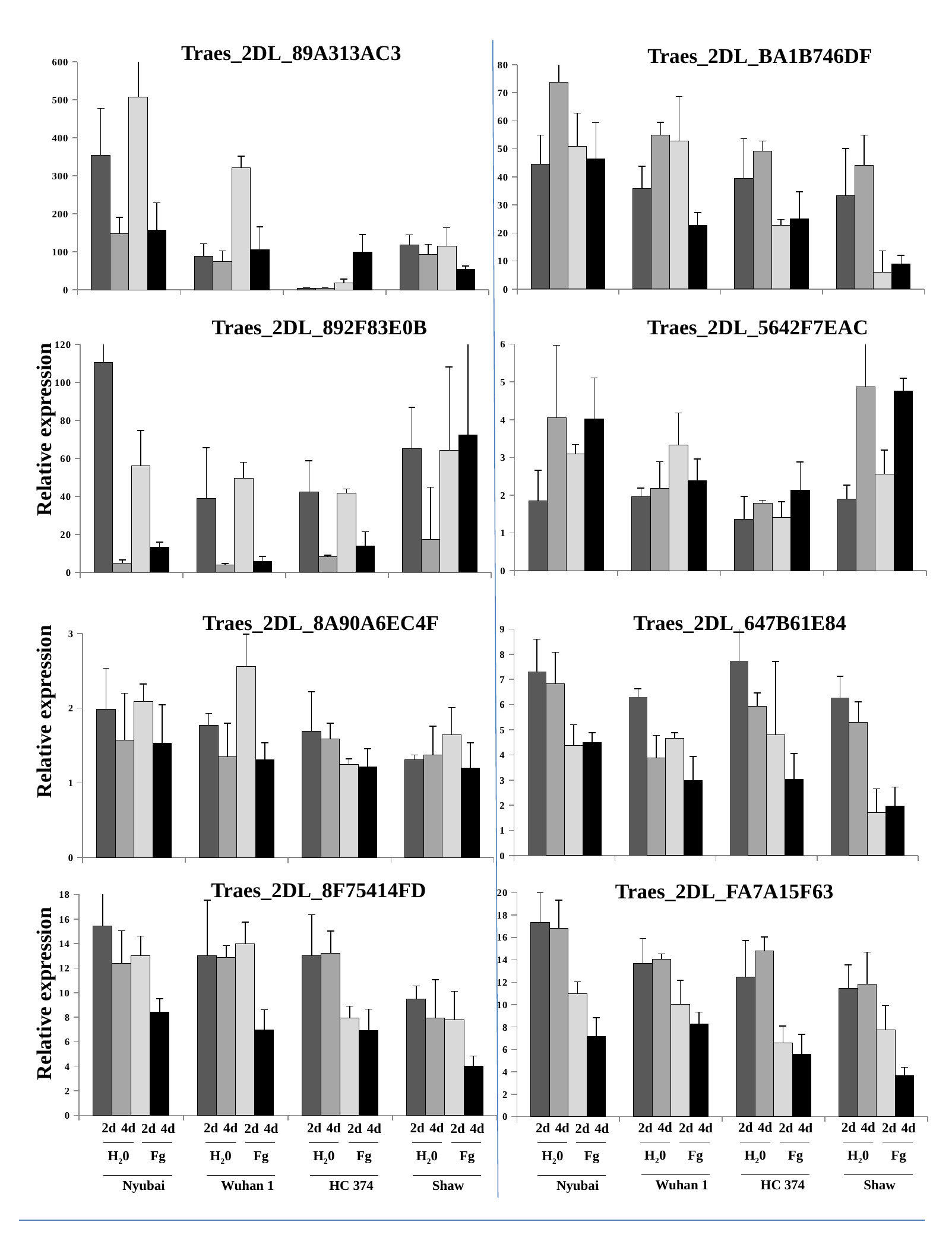

Traes_2DL_89A313AC3
Traes_2DL_BA1B746DF
### Chart
| Category | H20 2d | H20 4d | Fg 2d | Fg 4d |
|---|---|---|---|---|
| Nyubai | 44.54774109360577 | 73.74327913344788 | 50.95821571709043 | 46.37397467892566 |
| Wuhan | 35.86647261196268 | 54.86773413564969 | 52.8810345781388 | 22.793828371202405 |
| HC374 | 39.40082933683436 | 49.262676128825625 | 22.63514155445567 | 25.12349319078443 |
| Shaw | 33.375658576042596 | 44.06331287035126 | 6.069516148711066 | 8.88911792838958 |
### Chart
| Category | H20 2d | H20 4d | Fg 2d | Fg 4d |
|---|---|---|---|---|
| Nyubai | 354.70644214615226 | 147.13955394897894 | 507.8157767920249 | 156.4149927488049 |
| Wuhan | 88.33679180848087 | 74.54793807053817 | 321.0549950316848 | 104.99742974437952 |
| HC374 | 3.6278274802525527 | 3.5471675381395067 | 18.280710829408417 | 98.65771590893108 |
| Shaw | 117.7701653054922 | 92.24069969310358 | 114.59784109020525 | 54.67456515100755 |Traes_2DL_892F83E0B
Traes_2DL_5642F7EAC
### Chart
| Category | H20 2d | H20 4d | Fg 2d | Fg 4d |
|---|---|---|---|---|
| Nyubai | 1.8433789354420103 | 4.054765677226523 | 3.0923595625845794 | 4.026480335744167 |
| Wuhan | 1.9532372556080606 | 2.1804816275425636 | 3.3330614543383223 | 2.3904971545363405 |
| HC374 | 1.366153681968675 | 1.7875665734002995 | 1.4101497837858323 | 2.1275506069819774 |
| Shaw | 1.8953755958268232 | 4.862307819682207 | 2.5622495465630437 | 4.7503196561005945 |
### Chart
| Category | H20 2d | H20 4d | Fg 2d | Fg 4d |
|---|---|---|---|---|
| Nyubai | 110.44533113450275 | 4.7575487207476 | 56.228681024581384 | 13.33526212730637 |
| Wuhan | 38.813858586356695 | 3.7731357068842364 | 49.51885895154066 | 5.637894438441165 |
| HC374 | 42.35830910950121 | 8.236753119708382 | 41.55029823360922 | 13.934147975832571 |
| Shaw | 65.14301773864018 | 17.333379696967313 | 64.32292200016835 | 72.29762677553389 |Relative expression
### Chart
| Category | H20 2d | H20 4d | Fg 2d | Fg 4d |
|---|---|---|---|---|
| Nyubai | 1.9883102611675498 | 1.5715904501146813 | 2.0888601229721036 | 1.528799874449929 |
| Wuhan | 1.7738664251157477 | 1.346467934837418 | 2.5541727513800456 | 1.308221314851662 |
| HC374 | 1.6891657314274457 | 1.5909529313068251 | 1.2464911350167045 | 1.2157225710754125 |
| Shaw | 1.3065612925279606 | 1.3738505734226216 | 1.6469641121493792 | 1.1963096977735024 |Traes_2DL_8A90A6EC4F
Traes_2DL_647B61E84
### Chart
| Category | H20 2d | H20 4d | Fg 2d | Fg 4d |
|---|---|---|---|---|
| Nyubai | 7.3196223977165795 | 6.829786810700753 | 4.370174575685692 | 4.498927902476553 |
| Wuhan 1 | 6.306272050224457 | 3.879977358296627 | 4.650849315671931 | 2.9913444846480672 |
| HC374 | 7.728394512033738 | 5.922175852633232 | 4.795645945299767 | 3.038622088956256 |
| Shaw | 6.277808890712289 | 5.304489786668078 | 1.6970867058595183 | 1.9729478620409096 |Relative expression
### Chart
| Category | H20 2d | H20 4d | Fg 2d | Fg 4d |
|---|---|---|---|---|
| Nyubai | 15.447135812240239 | 12.402784933130318 | 12.997630797030993 | 8.437805646061316 |
| Wuhan | 13.022123276179373 | 12.865994012138463 | 13.998095073227452 | 6.952360337894106 |
| HC374 | 13.037948725145029 | 13.20439399822113 | 7.938275944731449 | 6.9370972350817945 |
| Shaw | 9.47026172318469 | 7.916562962154951 | 7.784411248083285 | 3.9876024917564195 |Traes_2DL_8F75414FD
Traes_2DL_FA7A15F63
### Chart
| Category | H20 2d | H20 4d | Fg 2d | Fg 4d |
|---|---|---|---|---|
| Nyubai | 17.36386115440866 | 16.82772818866602 | 10.960546859016608 | 7.16944145045517 |
| Wuhan | 13.70630222509181 | 14.038197491892808 | 10.019049237023749 | 8.297120597141513 |
| HC374 | 12.46911151433622 | 14.783372154673378 | 6.60579473499886 | 5.5776597919819 |
| Shaw | 11.458589820615837 | 11.84990044516156 | 7.72648275569989 | 3.6553242724326704 |Relative expression
4d
2d
4d
H20
Fg
Wuhan 1
2d
2d
4d
2d
4d
H20
Fg
HC 374
2d
4d
2d
4d
H20
Fg
Shaw
2d
4d
2d
4d
H20
Fg
Nyubai
2d
4d
2d
4d
H20
Fg
Wuhan 1
2d
4d
2d
4d
H20
Fg
HC 374
2d
4d
2d
4d
H20
Fg
Shaw
2d
4d
2d
4d
H20
Fg
Nyubai
