# Supplementary material for: Identification and mapping of expressed genes associated with the 2DL QTL for fusarium head blight resistance in the wheat line Wuhan 1
Source: BMC Genet. 2019 May 21;20:47. doi: 10.1186/s12863-019-0748-6 (PMC6528218; doi:10.1186/s12863-019-0748-6)
Supplement: Supplementary file 9 — FHB symptoms for 85 DH lines of the mapping population derived from Wuhan 1/Nyubai. Average percent infected florets per head after single floret inoculation, as described in Table 2. When known, presence (+) or absence (−) of the 2DL QTL for FHB resistance is indicated below the name of each NIL and the 2 parents, based on the presence of the Wuhan 1 or Nyubai allele for gwm539. (PPTX 71 kb) [file 12863_2019_748_MOESM9_ESM.pptx]

## Slide 1
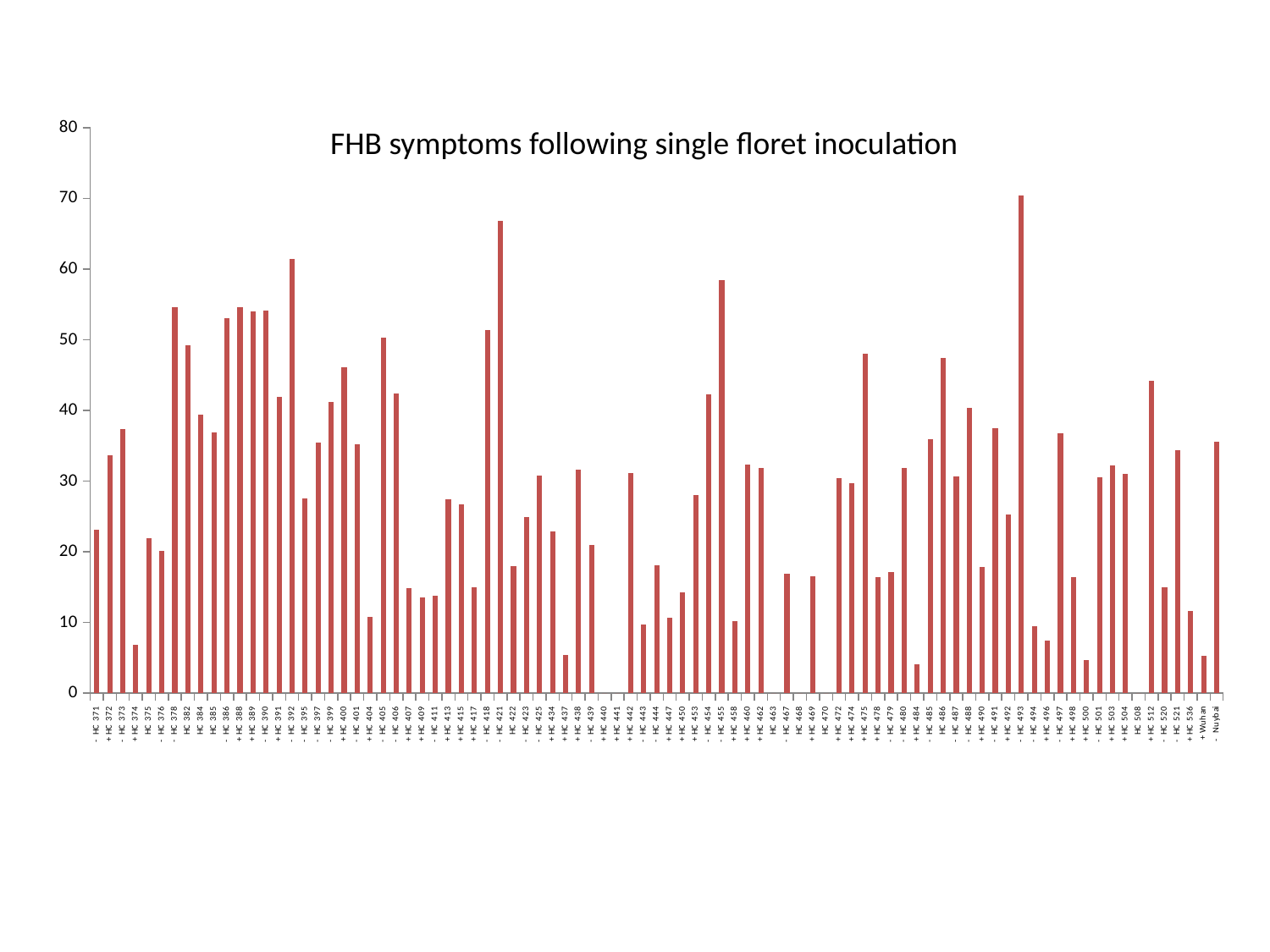

### Chart
| Category | FHB_sfi_mean from Curt |
|---|---|
| - HC 371 | 23.077581369248037 |
| + HC 372 | 33.60538615973399 |
| - HC 373 | 37.329434697855746 |
| + HC 374 | 6.8908285756111844 |
| HC 375 | 21.928258145363408 |
| - HC 376 | 20.172102865601314 |
| - HC 378 | 54.659048680787805 |
| HC 382 | 49.2479769685652 |
| HC 384 | 39.38517658525399 |
| HC 385 | 36.854288499025344 |
| - HC 386 | 53.004148629148624 |
| + HC 388 | 54.592482799004536 |
| + HC 389 | 54.02034125718336 |
| - HC 390 | 54.13000345065563 |
| + HC 391 | 41.979847494553375 |
| - HC 392 | 61.47527910685805 |
| - HC 395 | 27.54548591505113 |
| - HC 397 | 35.422522810293714 |
| - HC 399 | 41.15074190436509 |
| + HC 400 | 46.12786093049251 |
| - HC 401 | 35.2209004947008 |
| + HC 404 | 10.772058823529411 |
| - HC 405 | 50.36233073771774 |
| - HC 406 | 42.461788942052095 |
| + HC 407 | 14.874375853478021 |
| + HC 409 | 13.522588522588524 |
| - HC 411 | 13.832376916742245 |
| + HC 413 | 27.447576252723316 |
| + HC 415 | 26.688417325994727 |
| + HC 417 | 14.947013999645579 |
| - HC 418 | 51.39422172030867 |
| - HC 421 | 66.76843687713253 |
| HC 422 | 17.94871794871795 |
| - HC 423 | 24.944083694083695 |
| - HC 425 | 30.729000531632103 |
| + HC 434 | 22.935606060606062 |
| + HC 437 | 5.411123963755543 |
| + HC 438 | 31.58869395711501 |
| - HC 439 | 20.962905630088294 |
| + HC 440 | 0.0 |
| + HC 441 | 0.0 |
| + HC 442 | 31.15624027388733 |
| - HC 443 | 9.70811935885465 |
| - HC 444 | 18.053118908382068 |
| + HC 447 | 10.684518690517141 |
| + HC 450 | 14.324384192805244 |
| + HC 453 | 28.076032763532766 |
| - HC 454 | 42.322330447330444 |
| - HC 455 | 58.47282347282347 |
| + HC 458 | 10.215284944387111 |
| + HC 460 | 32.30848861283644 |
| + HC 462 | 31.80666638000229 |
| HC 463 | 0.0 |
| - HC 467 | 16.85294777686082 |
| HC 468 | 0.0 |
| + HC 469 | 16.56181379007466 |
| HC 470 | 0.0 |
| + HC 472 | 30.416666666666664 |
| + HC 474 | 29.734848484848488 |
| + HC 475 | 48.008685930512556 |
| + HC 478 | 16.430311890838205 |
| - HC 479 | 17.13249627336315 |
| - HC 480 | 31.906586800288842 |
| + HC 484 | 4.085648148148148 |
| - HC 485 | 35.93764519364365 |
| HC 486 | 47.47814685314685 |
| - HC 487 | 30.62251235077322 |
| - HC 488 | 40.36950963266752 |
| + HC 490 | 17.90430402930403 |
| - HC 491 | 37.50953159041394 |
| + HC 492 | 25.278361344537814 |
| - HC 493 | 70.3715034965035 |
| - HC 494 | 9.499878167641326 |
| + HC 496 | 7.4084358623832305 |
| - HC 497 | 36.71478532933641 |
| + HC 498 | 16.39462217635592 |
| + HC 500 | 4.678792569659443 |
| - HC 501 | 30.55143829600351 |
| + HC 503 | 32.20086200349358 |
| + HC 504 | 31.041666666666668 |
| HC 508 | 0.0 |
| + HC 512 | 44.22008547008547 |
| - HC 520 | 14.960311317509461 |
| - HC 521 | 34.32720057720058 |
| + HC 536 | 11.658399470899468 |
| + Wuhan | 5.3 |
| - Nuybai | 35.6 |FHB symptoms following single floret inoculation
