# Supplementary material for: Identification and mapping of expressed genes associated with the 2DL QTL for fusarium head blight resistance in the wheat line Wuhan 1
Source: BMC Genet. 2019 May 21;20:47. doi: 10.1186/s12863-019-0748-6 (PMC6528218; doi:10.1186/s12863-019-0748-6)
Supplement: Supplementary file 10 — Correlation between FHB symptoms following single floret inoculation (FHB_SFI) and expression of candidate genes in the DH population derived from Wuhan 1/Nyubai. (DOCX 23 kb) [file 12863_2019_748_MOESM10_ESM.docx]

**Additional file 10.** Correlation between FHB symptoms following single floret inoculation (FHB_SFI) and expression of candidate genes in the double haploid population derived from Wuhan 1/Nyubai.

| Candidate gene | Correlation with FHB_SFI |
| --- | --- |
| UN25696 | -0.071649547 |
| Traes_2DL_03CAAB80 | -0.184842606 |
| Traes_2DL_07F08C844 | -0.058499744 |
| Traes_2DL_179570792 | -0.099442195 |
| Traes_2DL_37D967CFC | 0.005073458 |
| Traes_2DL_382370E3B | -0.184842606 |
| Traes_2DL_A208876FE | -0.230404719 |
| Traes_2DL_B7ABC1CB9 | -0.055823998 |
