# Supplementary figures and images for: Identification and mapping of expressed genes associated with the 2DL QTL for fusarium head blight resistance in the wheat line Wuhan 1
Source: BMC Genet. 2019 May 21;20:47. doi: 10.1186/s12863-019-0748-6 (PMC6528218; doi:10.1186/s12863-019-0748-6)

## Slide 1
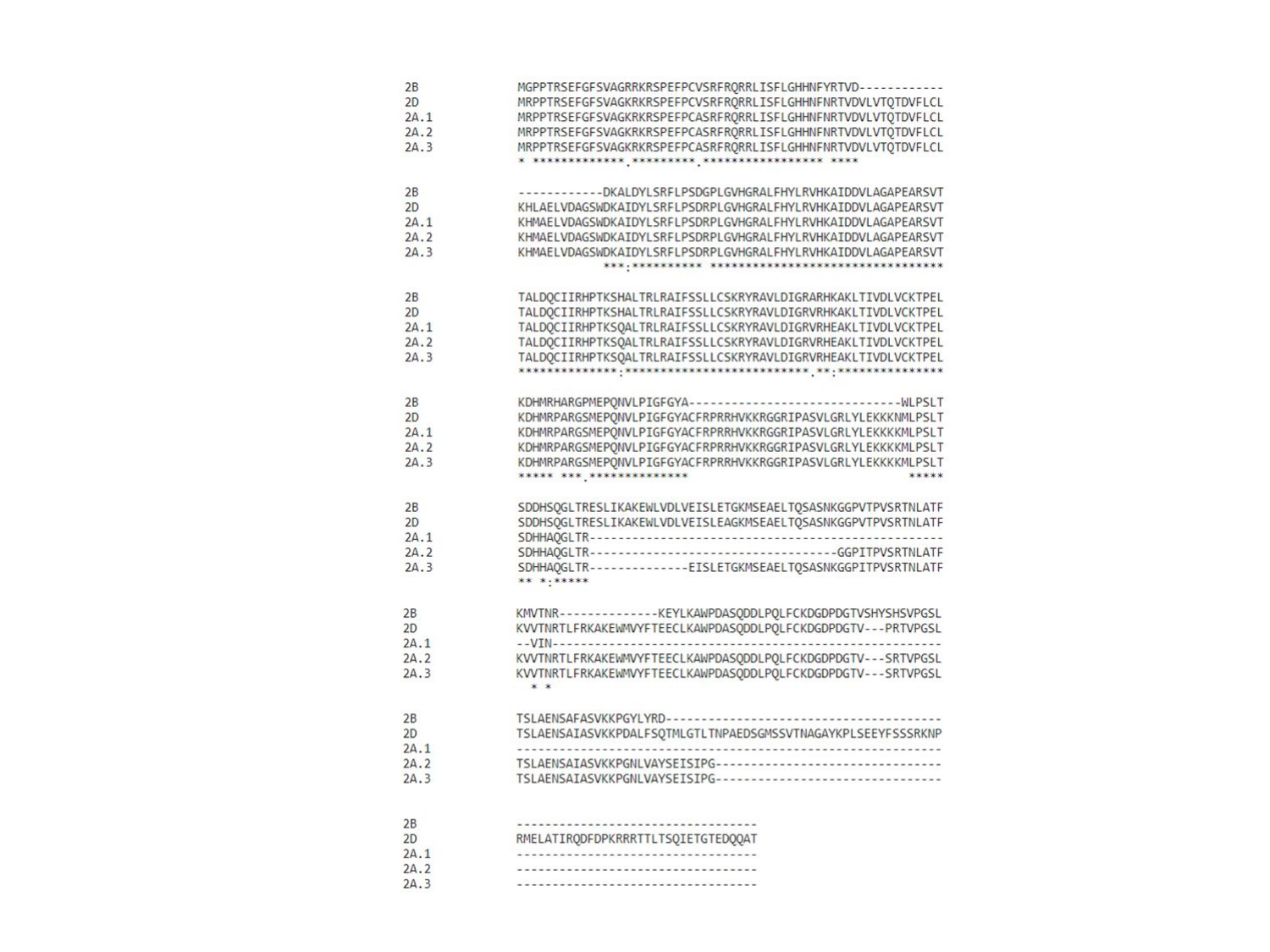

Supplement: Supplementary file 12 — Amino acid sequence alignment for Traes_2DL_179570792 and the homeologous genes on wheat genomes A and B. 2D, Traes_2DL_179570792; 2B, Traes_2BL_410E9E91D; 2A.1 to 2A.3, predicted protein isoforms of Traes_2AL_2079C6E79. Asterisks and dots under the aligned amino acids indicate homology between sequences. (PPTX 505 kb) [file 12863_2019_748_MOESM12_ESM.pptx]
