# Supplementary material for: Identification and mapping of expressed genes associated with the 2DL QTL for fusarium head blight resistance in the wheat line Wuhan 1
Source: BMC Genet. 2019 May 21;20:47. doi: 10.1186/s12863-019-0748-6 (PMC6528218; doi:10.1186/s12863-019-0748-6)
Supplement: Supplementary file 13 — Nucleotide (A) and amino acid (B) sequences alignment of the Unigene UN25696 (represented in the NCBI EST collection by the accession CD373927) with homeologous genomic sequences from chromosomes 2AL, 2BL and 2DL (as in IWGSC release 2.25). The arrows define the predicted borders of the coding sequence; the single base InDels are indicated by a black triangle; the larger InDel sequence in the B genome is boxed; the asterisks indicate premature stop codons. (PPTX 141 kb) [file 12863_2019_748_MOESM13_ESM.pptx]

## Slide 1
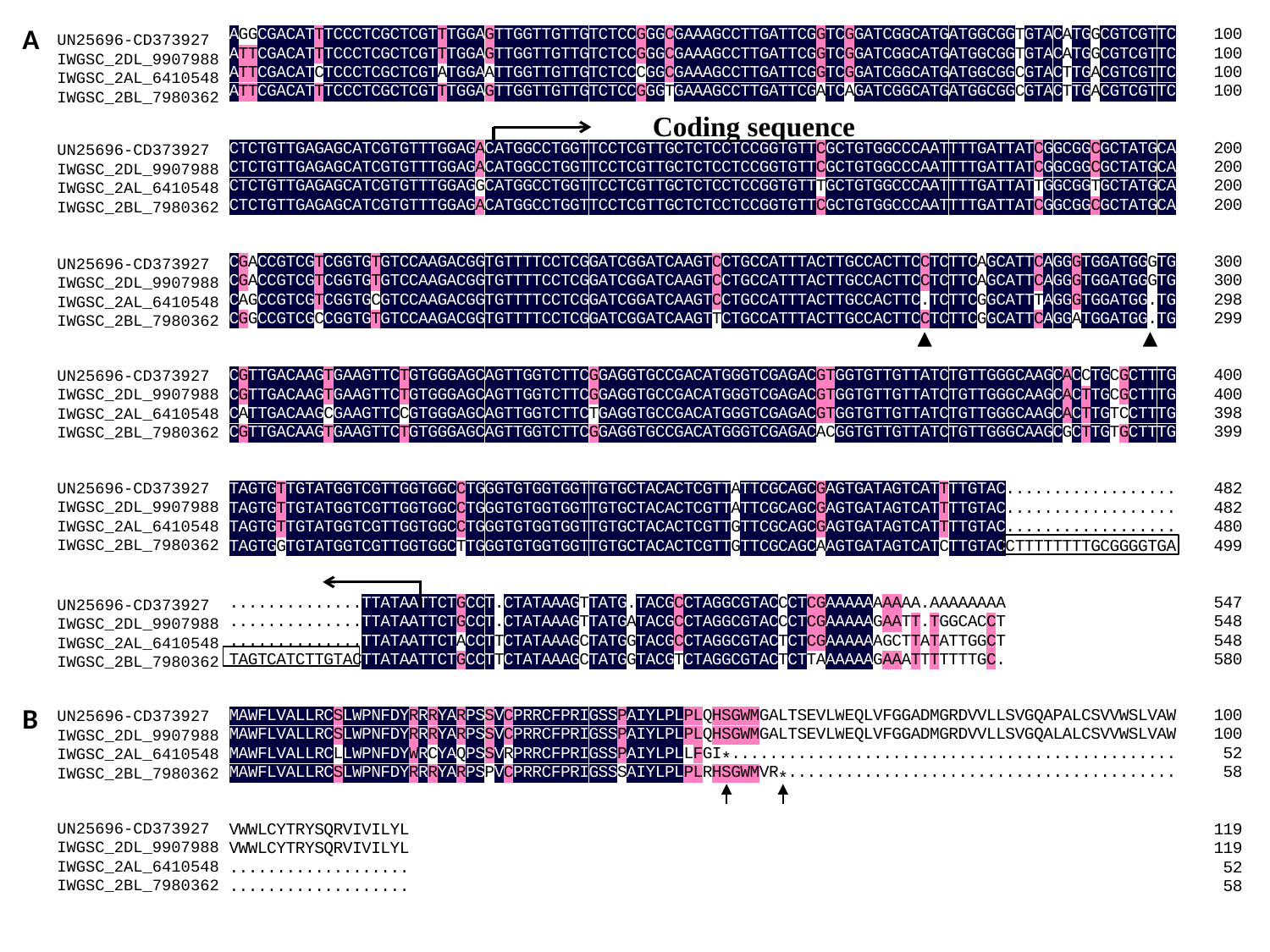

A
UN25696-CD373927
IWGSC_2DL_9907988
IWGSC_2AL_6410548
IWGSC_2BL_7980362
Coding sequence
UN25696-CD373927
IWGSC_2DL_9907988
IWGSC_2AL_6410548
IWGSC_2BL_7980362
UN25696-CD373927
IWGSC_2DL_9907988
IWGSC_2AL_6410548
IWGSC_2BL_7980362
UN25696-CD373927
IWGSC_2DL_9907988
IWGSC_2AL_6410548
IWGSC_2BL_7980362
UN25696-CD373927
IWGSC_2DL_9907988
IWGSC_2AL_6410548
IWGSC_2BL_7980362
UN25696-CD373927
IWGSC_2DL_9907988
IWGSC_2AL_6410548
IWGSC_2BL_7980362
B
UN25696-CD373927
IWGSC_2DL_9907988
IWGSC_2AL_6410548
IWGSC_2BL_7980362
*
*
UN25696-CD373927
IWGSC_2DL_9907988
IWGSC_2AL_6410548
IWGSC_2BL_7980362
